# Lactate Homeostasis Disruption Potentiates TACE‐Immunotherapy in Hepatocellular Carcinoma

**DOI:** 10.1002/advs.78484

**Published:** 2026-10-28

**Authors:** Minjiang Chen, Xiaoxiao Chen, Baozhu Yang, Hui Xu, Xueting Sun, Hongkai Qi, Huilin Hu, Ronghua Wu, Fengkai Qiu, Bing Wu, Yu Luo, Gaofeng Shu, Jiansong Ji

**Affiliations:** ^1^ Zhejiang Key Laboratory of Imaging and Interventional Medicine The Fifth Affiliated Hospital of Wenzhou Medical University Lishui China; ^2^ International Clinical School Hangzhou Medical College Lishui China; ^3^ Department of Radiology Lishui Central Hospital Lishui China; ^4^ Department of Pharmacy The Fifth Affiliated Hospital of Wenzhou Medical University Lishui China; ^5^ Shanghai Engineering Research Center of Pharmaceutical Intelligent Equipment Shanghai Frontiers Science Research Center For Druggability of Cardiovascular Non‐Coding RNA Institute For Frontier Medical Technology School of Chemistry and Chemical Engineering Shanghai University of Engineering Science Shanghai China

**Keywords:** acid–base homeostasis, embolic microspheres, hepatocellular carcinoma, lactate metabolism, transarterial chemoembolization

## Abstract

Developing strategies that simultaneously overcome chemoresistance and immune suppression remains a major challenge for improving transarterial chemoembolization (TACE) in hepatocellular carcinoma (HCC). Inspired by clinically used CaCO_3_ microspheres in TACE, we propose an “extracellular buffering‐intracellular metabolic disruption” strategy to precisely regulate lactate homeostasis for targeted therapy. Herein, we engineered multifunctional embolic microspheres (SDCa‐MSs) co‐loaded with calcium carbonate nanoparticles (CaNPs), syrosingopine (Syr), and doxorubicin (DOX) for TACE application. Within the acidic tumor microenvironment, SDCa‐MSs buffer extracellular acidity while disrupting intracellular lactate metabolism, thereby simultaneously remodeling extracellular and intracellular pH homeostasis. This coordinated regulation enhances DOX efficacy, induces calcium dyshomeostasis and immunogenic cell death, and ultimately stimulates robust antitumor immunity. In multiple murine HCC models, SDCa‐MSs markedly suppress tumor growth, remodel the tumor immune microenvironment, and synergistically enhance the therapeutic efficacy of anti‐PD‐L1 immunotherapy. By integrating metabolic regulation, chemotherapy, and immune activation into a single embolic platform, this work establishes an acid–base homeostasis‐regulating strategy for potentiating TACE and provides a conceptual framework for improving locoregional cancer immunotherapy.

## Graphical abstract

Lactate homeostasis is disrupted through spatially coordinated extracellular neutralization and intracellular acidification, triggering calcium overload, mitochondrial dysfunction, and immunogenic cell death. This metabolic reprogramming alleviates the immunosuppressive tumor microenvironment and enhances immune checkpoint blockade, providing a synergistic strategy to potentiate TACE–immunotherapy for hepatocellular carcinoma.

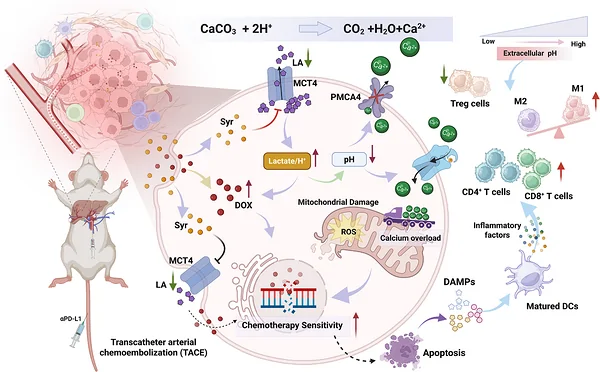

## 1 Introduction

Hepatocellular carcinoma (HCC) remains a global health challenge, ranking as the fourth most common cause of cancer‐related death worldwide, with over 900 000 new cases diagnosed annually [1, 2]. HCC is diagnosed at intermediate or advanced stages in over 70% of patients due to its asymptomatic early progression and rapid clinical deterioration [3]. Transarterial chemoembolization (TACE) has emerged as the cornerstone therapy for unresectable HCC [4, 5], combining ischemic necrosis through arterial embolization with localized chemotherapy to synergistically inhibit tumor growth [6, 7, 8, 9]. However, despite these advantages, patients with HCC undergoing TACE still experience a high rate of recurrence and metastasis [10].

A key hallmark of HCC is the “Warburg effect,” a metabolic shift toward aerobic glycolysis that results in excessive lactate production and extracellular acidification (pHe ≈6.0 vs. pHi ≈7.4) [11, 12, 13]. This acidic milieu promotes tumor progression by facilitating invasion, metastasis, angiogenesis, and immune evasion, while simultaneously reducing the efficacy of chemotherapy [14, 15, 16, 17]. Lactate accumulation stabilizes hypoxia‐inducible factor‐1 subunit alpha (HIF‐1α), upregulating monocarboxylate transporters (MCTs; [SLC16A1/MCT1] and [SLC16A3/MCT4]) to sustain glycolytic flux and further acidify the extracellular microenvironment [18, 19, 20, 21]. Consequently, the acidic tumor microenvironment (TME) suppresses antitumor immunity by impairing cytotoxic T lymphocyte (CTL) infiltration, promoting M2 macrophage polarization, and enhancing regulatory T‐cell activity [12]. Moreover, lactate serves as an oncometabolite, fueling oxidative phosphorylation in chemotherapy‐resistant cells and reducing the efficacy of chemotherapy. Consequently, strategies aimed at modifying the acidic TME have shown promise in enhancing the therapeutic response to TACE [22, 23].

Given the pivotal role of lactate‐driven acidosis in HCC progression, modulation of tumor acid–base homeostasis has emerged as a promising strategy for improving TACE outcomes. Indeed, a clinical trial demonstrated that neutralizing intratumoral lactic acid via infusion of a sodium bicarbonate (NaHCO_3_) solution significantly improved the response rate to TACE therapy [24]. However, the rapid diffusion and clearance of soluble buffering agents substantially limit the duration of pH regulation, highlighting the need for biomaterial‐based platforms capable of sustained remodeling of the acidic TME. Furthermore, current pH‐modulating approaches primarily focus on neutralizing extracellular acidity while largely overlooking the contribution of intracellular lactate accumulation and intracellular acid stress to tumor survival and therapeutic resistance [25]. Importantly, extracellular and intracellular pH are dynamically coupled but biologically distinct, suggesting that targeting only one compartment is insufficient to comprehensively reverse lactate‐driven tumor adaptation. Whether coordinated regulation of extracellular and intracellular acid–base homeostasis can simultaneously overcome lactate‐driven metabolic adaptation and restore antitumor immunity remains largely unexplored.

Among currently available pH‐responsive biomaterials, calcium carbonate nanoparticles (CaNPs) are particularly attractive because of their intrinsic proton‐buffering capacity, excellent biocompatibility, and calcium‐releasing properties under acidic conditions [9, 26, 27, 28]. By scavenging excess extracellular protons, CaNPs effectively alleviate tumor acidosis while simultaneously providing exogenous Ca^2+^. However, buffering extracellular acidity alone cannot interrupt the continuous intracellular production and export of lactate that sustains metabolic adaptation, indicating that durable metabolic reprogramming requires coordinated regulation of both extracellular and intracellular lactate homeostasis. Syrosingopine (Syr), a dual inhibitor of MCT1 and MCT4, effectively blocks lactate efflux, leading to intracellular lactate retention and sustained intracellular acidification [25, 29]. We therefore reasoned that coupling extracellular proton neutralization with intracellular lactate retention would establish a spatially coordinated regulation of tumor acid–base homeostasis capable of overcoming lactate‐driven metabolic adaptation. Such coordinated regulation has the potential to remodel both the extracellular tumor microenvironment and intracellular metabolic state, thereby perturbing calcium homeostasis, impairing mitochondrial bioenergetics, and ultimately sensitizing tumor cells to chemotherapy.

Herein, we developed a multifunctional embolic platform (SDCa‐MSs) by co‐encapsulating Syr, doxorubicin (DOX), and CaNPs within poly(lactic‐co‐glycolic acid) (PLGA) microspheres to establish a spatially coordinated strategy for regulating tumor lactate homeostasis. Following transarterial embolization, sustained release of CaNPs continuously neutralizes extracellular acidity, whereas Syr inhibits lactate efflux and promotes intracellular lactate retention, thereby coordinately remodeling extracellular and intracellular lactate homeostasis. This metabolic intervention disrupts intracellular calcium homeostasis, impairs mitochondrial function, enhances DOX‐induced cytotoxicity, and promotes immunogenic cell death (ICD). Consequently, SDCa‐MSs effectively remodel the immunosuppressive tumor immune microenvironment, transforming immunologically “cold” tumors into an inflamed, T‐cell‐infiltrated phenotype that exhibits remarkable synergy with anti‐PD‐L1 immune checkpoint blockade. Our findings establish spatially coordinated regulation of tumor acid–base homeostasis as a therapeutic principle for overcoming lactate‐driven chemoresistance and immune suppression, providing a conceptual framework for next‐generation locoregional immunotherapy in advanced HCC.

## 2 Results

### 2.1 Lactate Metabolism Drives HCC Progression and Associates With TACE Resistance

To investigate the role of lactate metabolism in HCC progression, we integrated bioinformatic analyses of public databases with validation using clinical specimens (Figure 1a). Analysis of The Cancer Genome Atlas (TCGA) dataset revealed significantly higher expression levels of the lactate transporters SLC16A1 (MCT1) (*p* = 0.0041) and SLC16A3 (MCT4) (*p* < 0.0001) in HCC tissues compared to adjacent non‐tumor liver parenchyma (Figure 1b,c). Elevated expression of either transporter was significantly associated with poorer overall survival, underscoring the clinical relevance of lactate transport in HCC progression (Figure 1d,e). These transcriptomic findings were further validated at the protein level by immunofluorescence staining of paired HCC and peritumoral liver tissues, which confirmed increased expression of LDHA, MCT1, and MCT4 in tumor tissues (Figure 1f). To further explore the potential link between lactate metabolism and TACE therapeutic resistance, we analyzed a transcriptomic dataset of patients with HCC from the Gene Expression Omnibus (GEO, GSE104580) stratified by their TACE response. Differential gene expression analysis between TACE responders and non‐responders revealed enrichment of metabolic pathways and carbon metabolism (Figure 1g,h). Consistent with these pathway alterations, several lactate metabolism‐related genes were differentially expressed between the two groups. LDHA and MCT4 were significantly upregulated in TACE non‐responders, whereas MCT1 exhibited an increasing trend without reaching statistical significance (Figure 1i). Collectively, these findings identify dysregulated lactate metabolism as a clinically relevant metabolic feature associated with HCC progression and poor response to TACE, supporting lactate transport as a potential therapeutic target.

**FIGURE 1:**
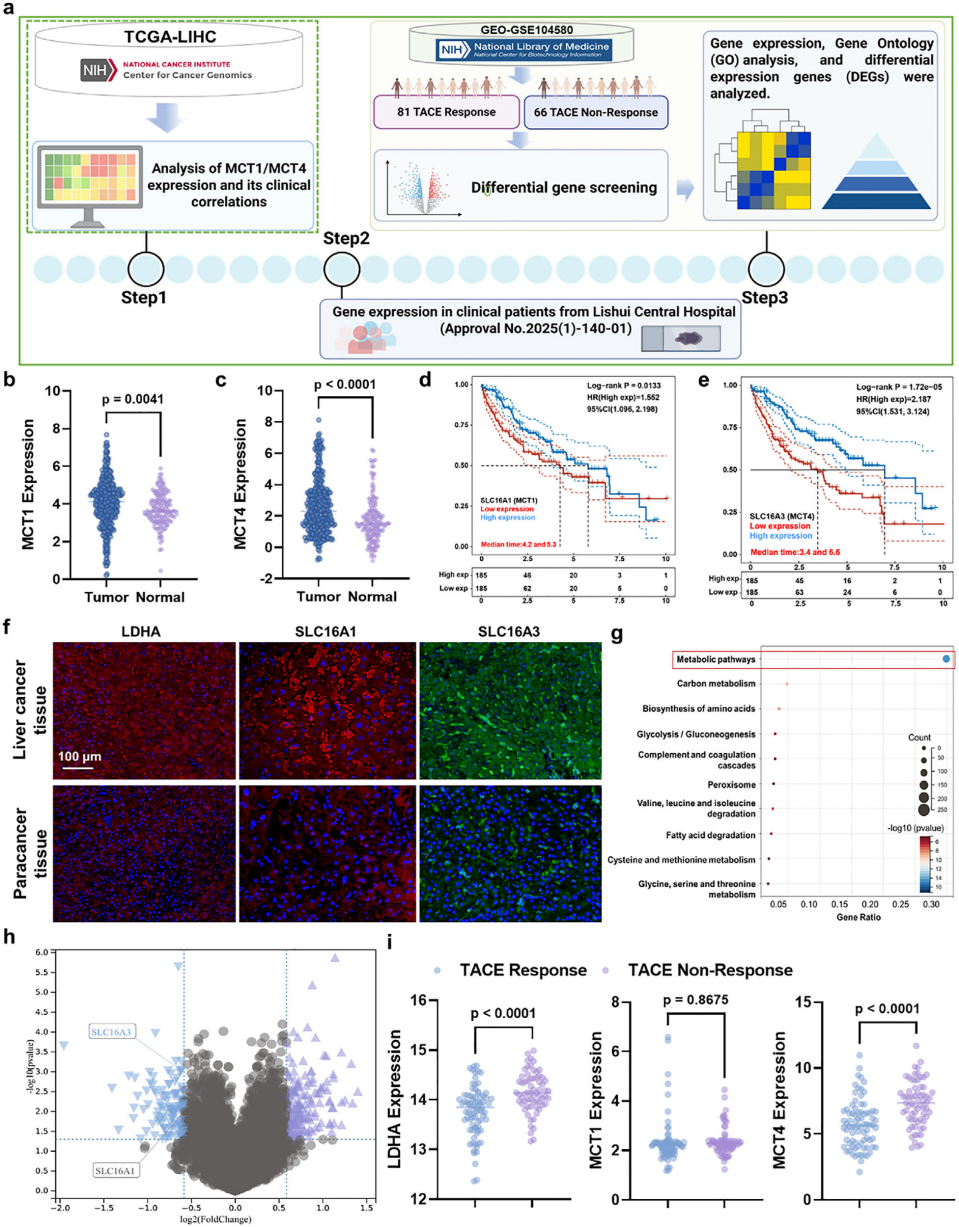
Lactate Metabolism Drives HCC Progression and Associates with TACE Resistance. (a) Schematic overview of the study design to investigate lactate metabolism in HCC progression and TACE resistance. (b, c) Analysis of the TCGA dataset revealed the significant upregulation of MCT1 (b) and MCT4 (c) expression in HCC tissues compared to adjacent non‐tumor liver tissues. (d, e) Kaplan‐Meier analysis of overall survival according to MCT1 (d) and MCT4 (e) expression. (f) Representative immunofluorescence images of LDHA, MCT1, and MCT4 expression in HCC and adjacent non‐tumor tissues. Scale bar: 100 µm. (g) KEGG pathway enrichment analysis of DEGs between TACE responders and non‐responders. (h) Volcano plot of DEGs between TACE responders and non‐responders. (i) Expression levels of LDHA, MCT1, and MCT4 in responders and non‐responders. Exact p‐values are shown in the corresponding panels.

### 2.2 Coordinated Regulation of Tumor Acid–Base Homeostasis Restores Chemosensitivity

To characterize the effects of coordinated regulation of tumor acid–base homeostasis on chemosensitivity, we first examined the effects of Syr and CaCO_3_ on lactate metabolism and pH homeostasis in Hepa1‐6 cells. CaCO_3_ treatment reduced extracellular lactate accumulation, whereas Syr promoted intracellular lactate retention by inhibiting lactate export (Figure 2a,b). These distinct metabolic effects were accompanied by differential regulation of pH. CaCO_3_ effectively neutralized extracellular acidity, increasing the medium pH to 7.14 and 7.25 at 90 min when administered alone or in combination with Syr, respectively (Figure 2c). In contrast, Syr markedly decreased intracellular pH to approximately 5.85, whereas CaCO_3_ maintained intracellular pH near the physiological range (approximately 7.13) (Figure 2d; Figure S1). The restoration of extracellular pH together with intracellular acidification prompted us to examine whether coordinated regulation of tumor acid–base homeostasis could improve chemotherapy responsiveness [15, 30, 31]. Confocal microscopy and flow cytometry demonstrated substantially increased intracellular accumulation of DOX following treatment with Syr and CaCO_3_, indicating enhanced intracellular and nuclear drug retention (Figure 2e,f). Consistently, exogenous lactic acid markedly increased the IC_50_ of DOX (13.4 ± 1.1 vs. 2.8 ± 0.4 µg·mL^−1^), whereas treatment with either Syr or CaCO_3_ partially restored DOX sensitivity. The combination treatment produced the greatest reduction in IC_50_ (1.43 ± 0.16 µg·mL^−1^) (Figure 2g).

**FIGURE 2:**
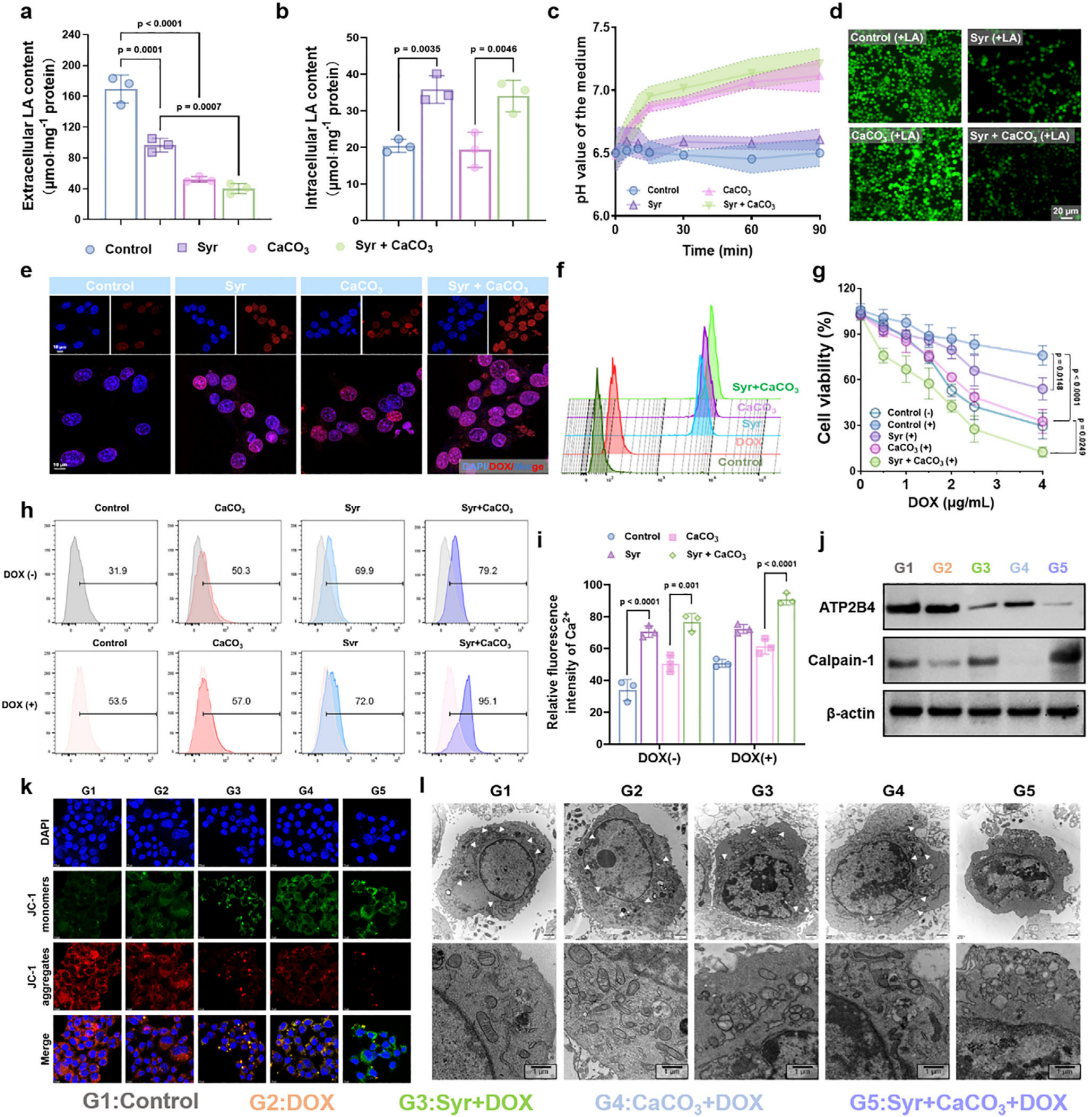
Coordinated Regulation of Tumor Acid–Base Homeostasis Restores Chemosensitivity. (a, b) Extracellular (a) and intracellular (b) lactate levels in Hepa1‐6 cells following different treatments. (c) Extracellular pH changes after different treatments. (d) Representative BCECF‐AM fluorescence images showing intracellular pH. Scale bar, 20 µm. (e) Representative CLSM images of intracellular DOX uptake under lactate conditions. Scale bar, 10 µm. (f) Flow cytometric quantification of intracellular DOX fluorescence. (g) Cell viability of Hepa1‐6 cells after different treatments. (+) and (−) indicate the presence or absence of exogenous lactate, respectively. (h) Representative flow cytometric histograms of intracellular Ca^2+^ using Fluo‐4 AM. (i) Quantification of intracellular Ca^2+^ fluorescence intensity. (j) Western blot analysis of ATP2B4 and Calpain‐1 expression. (k) Representative JC‐1 fluorescence images of mitochondrial membrane potential. Scale bar, 7.5 µm. (l) Representative TEM images of Hepa1‐6 cells after different treatments. White arrowheads indicate mitochondrial swelling, cristae disruption, and vacuolization. Data are presented as mean ± SD (*n* = 3 for (a–c, f, i); *n* = 6 for (g)). Statistical analyses were performed as described in the Experimental Section. Exact p‐values are indicated in the corresponding panels.

We next examined whether intracellular acidification induced by this strategy affected calcium homeostasis. Fluo‐4 AM staining showed that Syr markedly increased intracellular Ca^2+^ levels, whereas CaCO_3_ alone caused only modest changes. Combined treatment with Syr and CaCO_3_ further amplified Ca^2+^ accumulation (Figure 2h,i; Figure S2). A similar pattern was observed in the presence of DOX, with the highest intracellular Ca^2+^ level detected in the combination group. Consistent with these findings, co‐treatment increased Calpain‐1 expression while reducing ATP2B4 expression, indicating disruption of intracellular calcium homeostasis (Figure 2j). Disruption of calcium homeostasis was accompanied by pronounced mitochondrial injury. JC‐1 staining demonstrated a marked loss of mitochondrial membrane potential (ΔΨm) following combined treatment (Figure 2k; Figure S3), and transmission electron microscopy (TEM) revealed mitochondrial swelling, cristae disruption, and vacuolization (Figure 2l). These structural alterations were further accompanied by intracellular ATP depletion and increased ROS production (Figures S4 and S5). Collectively, these results demonstrate that coordinated regulation of tumor acid–base homeostasis induces calcium dyshomeostasis‐associated mitochondrial dysfunction, thereby restoring chemosensitivity and potentiating DOX‐induced cytotoxicity in HCC cells.

### 2.3 Preparation and Characterization of SDCa‐MSs

CaCO_3_ nanoparticles (CaNPs) were first synthesized via a gas diffusion method, yielding uniformly dispersed spherical nanoparticles with an average diameter of 100 ± 15.3 nm, as confirmed by TEM (Figure 3a,b). The CaNPs were subsequently co‐encapsulated with Syr and DOX into PLGA microspheres using an optimized emulsion‐solvent evaporation method to generate SDCa‐MSs (Figure 3c). For comparison, Syr and DOX were co‐encapsulated in PLGA microspheres without CaNPs to generate SD‐MSs. Scanning electron microscopy (SEM) demonstrated that both SD‐MSs and SDCa‐MSs exhibited smooth spherical morphology with comparable mean diameters of 47.1 ± 8.5 and 45.2 ± 10.8 µm, respectively, indicating that incorporation of CaNPs did not affect microsphere morphology or size distribution (Figure 3d; Figure S6). Stable particle size distributions were maintained throughout 7 days of storage, confirming favorable physicochemical stability (Figure S7). Successful fabrication of SDCa‐MSs was further verified by complementary physicochemical characterization. UV–vis spectroscopy identified the characteristic absorption peaks of Syr (260 and 295 nm) and DOX (480 nm), consistent with those of the corresponding free drugs (Figure 3e). Fourier‐transform infrared (FTIR) spectra revealed the characteristic absorption bands of PLGA (1742 cm^−1^), DOX (3100–3500 and 3650–3600 cm^−1^), and Syr (1675–1640 cm^−1^), confirming successful incorporation of both drugs into the microspheres (Figure 3f). Elemental mapping demonstrated homogeneous distribution of calcium (Ca), oxygen (O), carbon (C), and nitrogen (N) throughout the SDCa‐MSs (Figure S8), further confirming successful integration of all functional components.

**FIGURE 3:**
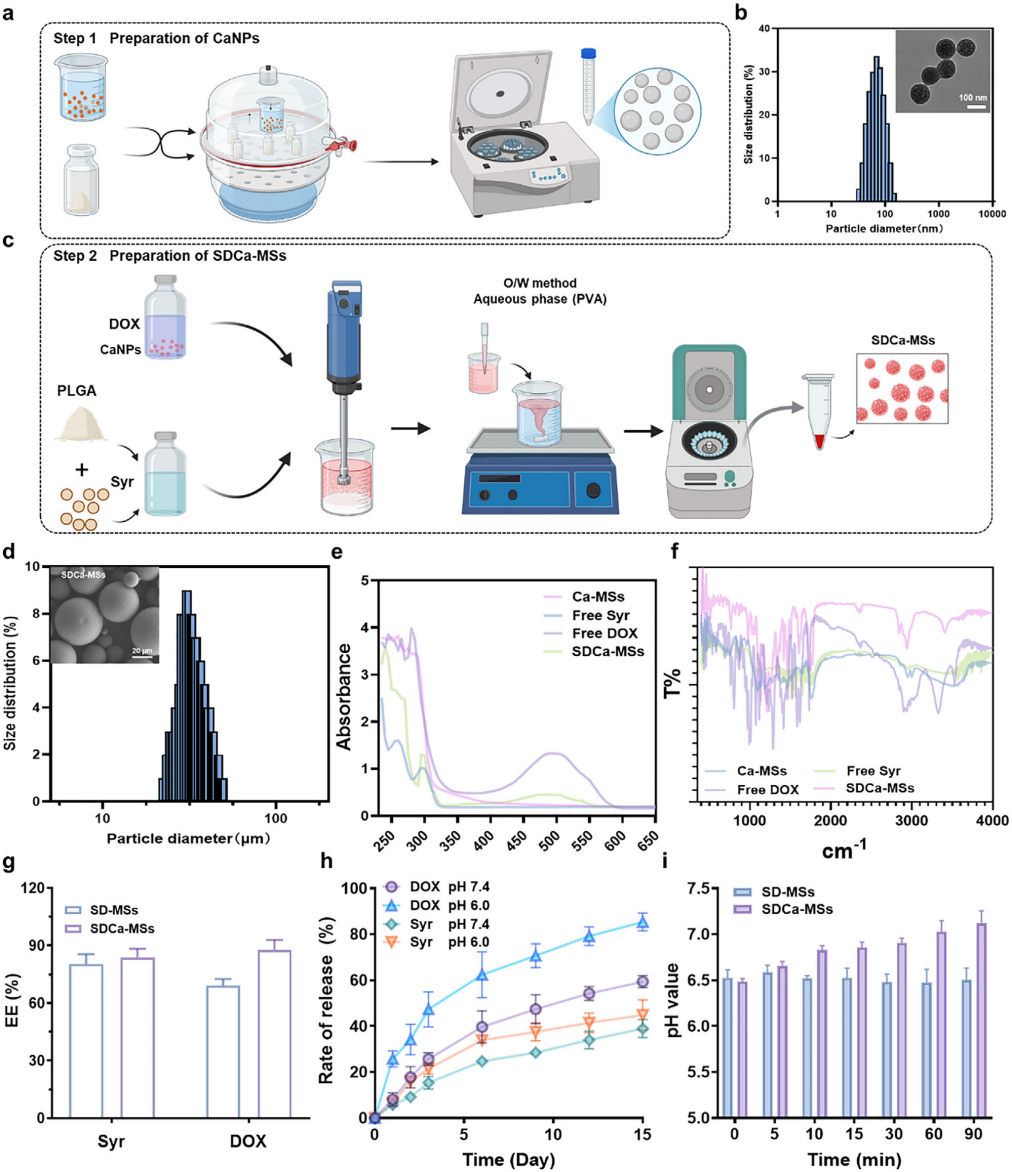
Preparation and Characterization of SDCa‐MSs. (a) Schematic illustration of the synthesis of CaNPs (Step 1). (b) Particle size distribution and representative TEM image of CaNPs. (c) Schematic illustration of the synthesis of SDCa‐MSs (Step 2). (d) Particle size distribution and representative SEM image of SDCa‐MSs. (e) UV–vis absorbance spectra of free Syr, free DOX, Ca‐MSs, and SDCa‐MSs. (f) FTIR spectra of free Syr, free DOX, Ca‐MSs, and SDCa‐MSs. (g) Drug EE of Syr and DOX in SD‐MSs and SDCa‐MSs. (h) In vitro release profiles of Syr and DOX from SDCa‐MSs under different pH conditions (pH 6.0 and 7.4). (i) Time‐dependent acid‐neutralizing capacity of SDCa‐MSs at an initial pH of 6.5. Data are presented as mean ± SD (*n* = 3). Statistical analyses were performed as described in the Experimental Section. Exact p‐values are indicated in the corresponding panels.

The drug loading (DL) and encapsulation efficiency (EE) of SDCa‐MSs were 1.72 ± 0.20% and 83.7 ± 4.5% for Syr, and 0.90 ± 0.24% and 87.7 ± 5.3% for DOX, respectively. SD‐MSs exhibited comparable Syr loading (DL: 1.83 ± 0.10%; EE: 80.2 ± 5.2%), but lower DOX loading and encapsulation efficiency (DL: 0.79 ± 0.20%; EE: 68.9 ± 3.5%) (Figure 3g and Figure S9). These results indicate that incorporation of CaNPs did not impair drug loading and improved DOX encapsulation efficiency. The release characteristics and buffering capacity of SDCa‐MSs were subsequently evaluated. DOX exhibited pronounced pH‐responsive release, with substantially accelerated release under acidic conditions (pH 6.0), whereas the release profile of Syr remained largely unaffected by pH (Figure 3h). This difference may reflect the distinct physicochemical properties of DOX and Syr, resulting in different release behaviors from the PLGA matrix under acidic conditions. Compared with SD‐MSs, incorporation of CaNPs further enhanced the acid responsiveness of DOX release while exerting minimal influence on Syr release (Figure S10). More importantly, SDCa‐MSs rapidly neutralized acidic environments under multiple acidic conditions. At an initial pH of 6.5, SDCa‐MSs elevated the buffer pH to approximately physiological neutrality (≈7.2) within 90 min. A similar buffering effect was observed under the more acidic condition of pH 6.0, whereas SD‐MSs exhibited negligible acid‐neutralizing capacity under either condition (Figure 3i and Figure S11). Collectively, these findings demonstrate that SDCa‐MSs combine robust acid‐neutralizing capability with pH‐responsive drug release, providing the physicochemical basis for coordinated regulation of tumor acid–base homeostasis during locoregional HCC therapy.

### 2.4 SDCa‐MSs Trigger ICD‐Driven Antitumor Immune Activation in Vitro

The antitumor activity of SDCa‐MSs was first evaluated in Hepa1‐6 cells. MTT assays showed that SDCa‐MSs exhibited the strongest cytotoxicity among all treatment groups (Figure S12). Flow cytometric analysis further demonstrated that SDCa‐MSs markedly increased apoptotic cell death, with an apoptosis rate of 68.1 ± 4.6%, compared with 28.1 ± 5.6% in cells treated with DOX‐loaded PLGA microspheres (D‐MSs) (Figure S13). Live/Dead staining produced consistent results, showing that SDCa‐MSs effectively overcame lactate‐associated chemoresistance and substantially enhanced tumor cell death (Figure S14). To determine whether this enhanced cytotoxicity was accompanied by ICD, representative damage‐associated molecular patterns (DAMPs) were evaluated. SDCa‐MSs significantly promoted extracellular ATP and HMGB1 release while increasing cell surface exposure of calreticulin (CRT), indicating efficient induction of ICD and generation of the canonical “find‐me” and “eat‐me” signals required for dendritic cell (DC) activation (Figure S15). The immunostimulatory consequences of ICD were subsequently assessed using a ternary co‐culture system comprising SDCa‐MSs‐treated tumor cells, bone marrow‐derived dendritic cells (BMDCs), and splenic lymphocytes (Figure S16). Flow cytometric analysis demonstrated that SDCa‐MSs markedly promoted BMDC maturation, as evidenced by significantly increased expression of the co‐stimulatory molecules CD80 and CD86 compared with all other groups (Figure S17). Enhanced DC maturation was accompanied by robust T‐cell activation, with 2.4‐ and 3.9‐fold increases in CD3^+^CD8^+^ cytotoxic T cells and CD3^+^CD4^+^ helper T cells, respectively, relative to the control group (Figure S18). In parallel, secretion of the effector cytokines TNF‐α and IFN‐γ was significantly elevated following SDCa‐MSs treatment (Figure S19). Together, these results demonstrate that SDCa‐MSs efficiently induce ICD and subsequently promote DC maturation, T‐cell activation, and antitumor immune responses in vitro.

### 2.5 In Vivo Antitumor Efficacy and TME Remodeling by SDCa‐MSs

Biodistribution studies in Hepa1‐6 tumor‐bearing mice revealed enhanced tumor accumulation and prolonged retention of ICG@Ca‐MSs compared with ICG‐MSs, with substantially stronger fluorescence signals maintained throughout the observation period (Figure S20). This prolonged retention is attributable to the incorporation of CaNPs, which continuously neutralize the acidic tumor microenvironment while preserving the structural integrity of the microspheres, thereby facilitating sustained intratumoral retention and controlled release of the encapsulated ICG. Based on these favorable biodistribution characteristics, the antitumor efficacy of SDCa‐MSs was subsequently evaluated according to the treatment schedule shown in Figure 4a. SDCa‐MSs achieved the greatest inhibition of tumor growth (79.3 ± 4.2%), significantly outperforming both single‐agent formulations (D‐MSs: 47.9 ± 8.4%; SCa‐MSs: 20.0 ± 7.1%) and combination controls (DCa‐MSs: 62.9 ± 7.5%; SD‐MSs: 61.7 ± 6.1%) (Figure 4b,c,e). No obvious systemic toxicity was observed during treatment, as reflected by stable body weights across all groups (Figure 4f). Intratumoral pH was subsequently quantified using the SNARF‐4F fluorescent probe. SDCa‐MSs effectively normalized tumor acidity, increasing the intratumoral pH to 7.09 ± 0.07, compared with 6.64 ± 0.11 in the SD‐MSs group (Figure 4d). SDCa‐MSs significantly reduced intratumoral lactate content, indicating effective alleviation of lactate accumulation within the tumor microenvironment (Figure S21). Fluo‐3 AM staining revealed markedly increased intratumoral Ca^2+^ accumulation following SDCa‐MSs treatment, whereas only minimal changes were observed in the control groups (Figure S22). Correspondingly, DHE staining demonstrated markedly increased intratumoral ROS levels following SDCa‐MSs treatment (Figure S23). Immunofluorescence staining further showed enhanced CRT exposure together with reduced nuclear HMGB1 staining, indicating robust induction of ICD in vivo (Figure S24). Histopathological analyses, including H&E, TUNEL, and Ki‐67 staining, revealed extensive tumor necrosis and apoptosis accompanied by markedly reduced tumor cell proliferation in the SDCa‐MSs group (Figure S25). Together, these results demonstrate that coordinated regulation of tumor acid–base homeostasis induces calcium dyshomeostasis‐associated oxidative stress and immunogenic cell death in vivo, contributing to the superior antitumor efficacy of SDCa‐MSs.

**FIGURE 4:**
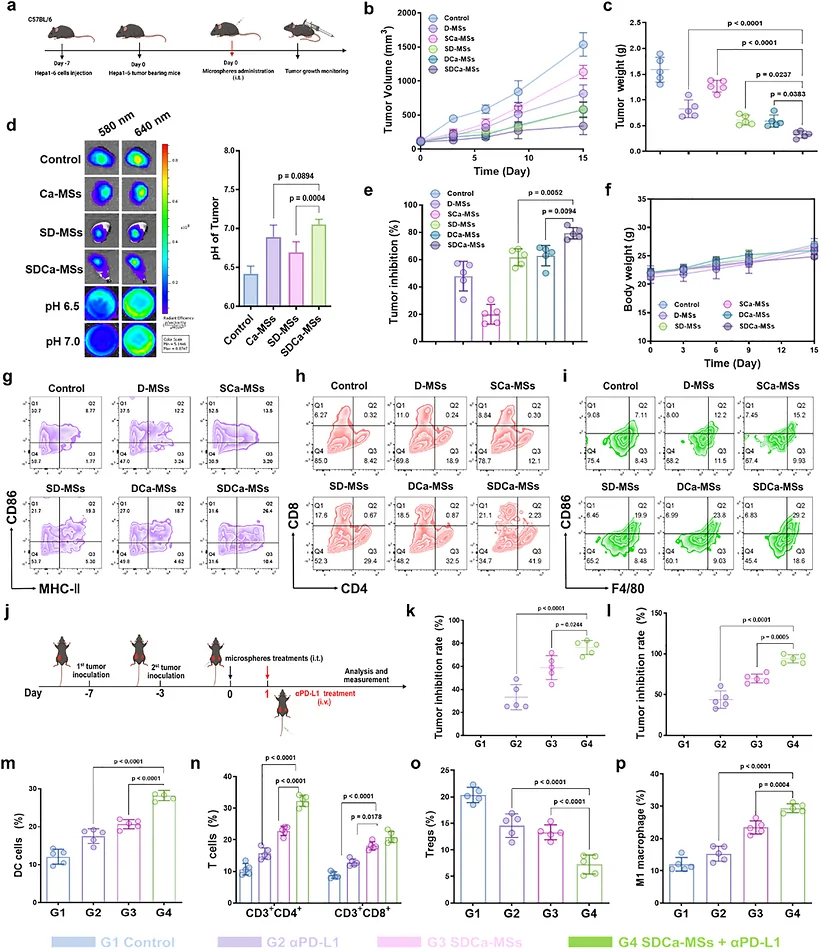
SDCa‐MSs Remodel the Tumor Immune Microenvironment and Potentiate Local and Systemic Antitumor Immunity. (a) Schematic illustration of the treatment protocol in the Hepa1‐6 subcutaneous tumor model. (b) Tumor growth curves of mice receiving different treatments. (c) Tumor weights in each group following different treatments. (d) Representative SNARF‐4F fluorescence images and intratumoral pH measurements following different treatments. Intratumoral pH was determined from the fluorescence emission ratio of SNARF‐4F at 580 and 640 nm and converted to pH values using a standard calibration curve. (e) Tumor inhibition rates in different treatment groups. (f) Body weight changes of mice during the treatment period. (g) Flow cytometric analysis of mature DCs (CD11c^+^MHC‐II^+^CD86^+^) in the tumor. (h, i) Flow cytometric analysis of tumor‐infiltrating T cells (CD3^+^CD4^+^ and CD3^+^CD8^+^) (h) and M1 macrophages (CD11b^+^F4/80^+^CD86^+^) (i). (j) Schematic illustration of the treatment protocol for the bilateral Hepa1‐6 tumor model. (k, l) Inhibition rates of the primary (k) and distant (l) tumors following different treatments. (m) Flow cytometric analysis of splenic mature DCs (CD11c^+^CD80^+^CD86^+^) in mice from the bilateral tumor model. (n–p) Flow cytometric analysis of T cells (n), Tregs (o), and M1 macrophages (p) in distant tumors following different treatments. Data are presented as mean ± SD (*n* = 5). Statistical analyses were performed as described in the Experimental Section. Exact p‐values are indicated in the corresponding panels.

The effects of SDCa‐MSs on the tumor immune microenvironment were next investigated. Flow cytometric analysis revealed increased infiltration of CD11c^+^MHC‐II^+^ DCs and an increased proportion of CD11c^+^MHC‐II^+^ CD86^+^ mature DCs following SDCa‐MSs treatment (Figure 4g; Figure S26). Mature DCs were likewise increased in the spleen (Figure S27). Consistent with enhanced DC activation, tumors treated with SDCa‐MSs exhibited substantial increases in both CD3^+^CD4^+^ helper T cells and CD3^+^CD8^+^ cytotoxic T cells (Figure 4h; Figure S28), whereas the proportion of Foxp3^+^CD25^+^ Treg cells was significantly reduced (Figure S29). Remodeling of the myeloid compartment was further characterized by increased infiltration of M1 macrophages together with a marked reduction in M2 macrophages (Figure 4i; Figures S30 and S31). These cellular alterations were accompanied by significantly elevated intratumoral levels of IFN‐γ and TNF‐α (Figure S32). Collectively, these findings demonstrate that SDCa‐MSs coordinate innate and adaptive immune activation, leading to extensive remodeling of the tumor immune microenvironment.

### 2.6 In Vivo Systemic Antitumor Immunity Induced by SDCa‐MSs Combined With αPD‐L1

The therapeutic efficacy of combining SDCa‐MSs with immune checkpoint blockade was evaluated using a bilateral Hepa1‐6 tumor model (Figure 4j). Treatment of the primary tumor with SDCa‐MSs alone produced a pronounced abscopal effect, resulting in significant inhibition of both primary and untreated distant tumors. αPD‐L1 monotherapy produced only modest tumor suppression, with inhibition rates of 33.2% and 43.8% in the primary and distant tumors, respectively. In comparison, SDCa‐MSs alone achieved substantially greater inhibition of both primary (58.9%) and distant (69.7%) tumors. The combination of SDCa‐MSs and αPD‐L1 produced the strongest therapeutic response, with tumor inhibition rates reaching 76.4% in the primary tumors and 93.8% in the distant tumors (Figure 4k,l; Figure S33). To characterize the systemic immune response induced by combination therapy, immune cell populations in the spleen and distant tumors were analyzed. Flow cytometric analysis revealed a marked increase in mature DCs following combination treatment (28.4% vs. 12.9% in the control group) (Figure 4m). This was accompanied by significant expansion of both CD3^+^CD4^+^ and CD3^+^CD8^+^ T‐cell populations (Figure 4n), together with a pronounced reduction in Tregs (Figure 4o). In addition, tumor‐associated macrophages displayed enhanced M1 polarization following combination therapy (Figure 4p; Figure S34). Together, these results demonstrate that SDCa‐MSs markedly potentiate the therapeutic efficacy of αPD‐L1 by promoting systemic antitumor immune activation.

### 2.7 CyTOF Reveals Comprehensive Remodeling of the Tumor Immune Landscape

To comprehensively profile the immune landscape following SDCa‐MSs‐based therapy, high‐dimensional single‐cell CyTOF analysis was performed on tumor tissues collected from different treatment groups (Figure 5a). A 40‐marker antibody panel was used to characterize the phenotypic and functional features of tumor‐infiltrating immune cells (Table S2). Heatmap visualization of normalized marker expression identified distinct lineage‐specific expression patterns across immune cell clusters (Figure 5b). Dimensionality reduction by t‐distributed stochastic neighbor embedding (t‐SNE) resolved CD45^+^ immune cells into 27 phenotypically distinct clusters, which were subsequently classified into nine major immune cell populations according to canonical lineage markers (Figure 5c). Representative immune populations, including T cells, DCs, Tregs, macrophages, neutrophils, B cells, and natural killer (NK) cells, were visualized on the corresponding t‐SNE maps (Figure S35). CyTOF analysis further demonstrated extensive remodeling of the tumor immune landscape following treatment with SDCa‐MSs combined with αPD‐L1. The abundance and distribution of multiple immune cell populations, including T cells, DCs, TAMs, and B cells, differed markedly from those of the untreated control group (Figure 5d). Combination therapy increased the proportions of both CD8^+^ cytotoxic T cells and CD4^+^ T cells (Figure 5e,f,i,j), together with an increased abundance of DCs (Figure 5g,k). The proportion of B cells was also elevated following combination treatment (Figure S36). In contrast, Tregs were significantly reduced (Figure 5h,l). Remodeling of the myeloid compartment was further characterized by enrichment of M1‐like TAMs (Figure 5m; Figure S37), whereas NK cells showed an increasing trend that did not reach statistical significance (Figure 5n; Figure S38). Together, these results demonstrate that SDCa‐MSs combined with αPD‐L1 extensively remodel the tumor immune landscape by promoting multiple antitumor immune cell populations while reducing immunosuppressive components.

**FIGURE 5:**
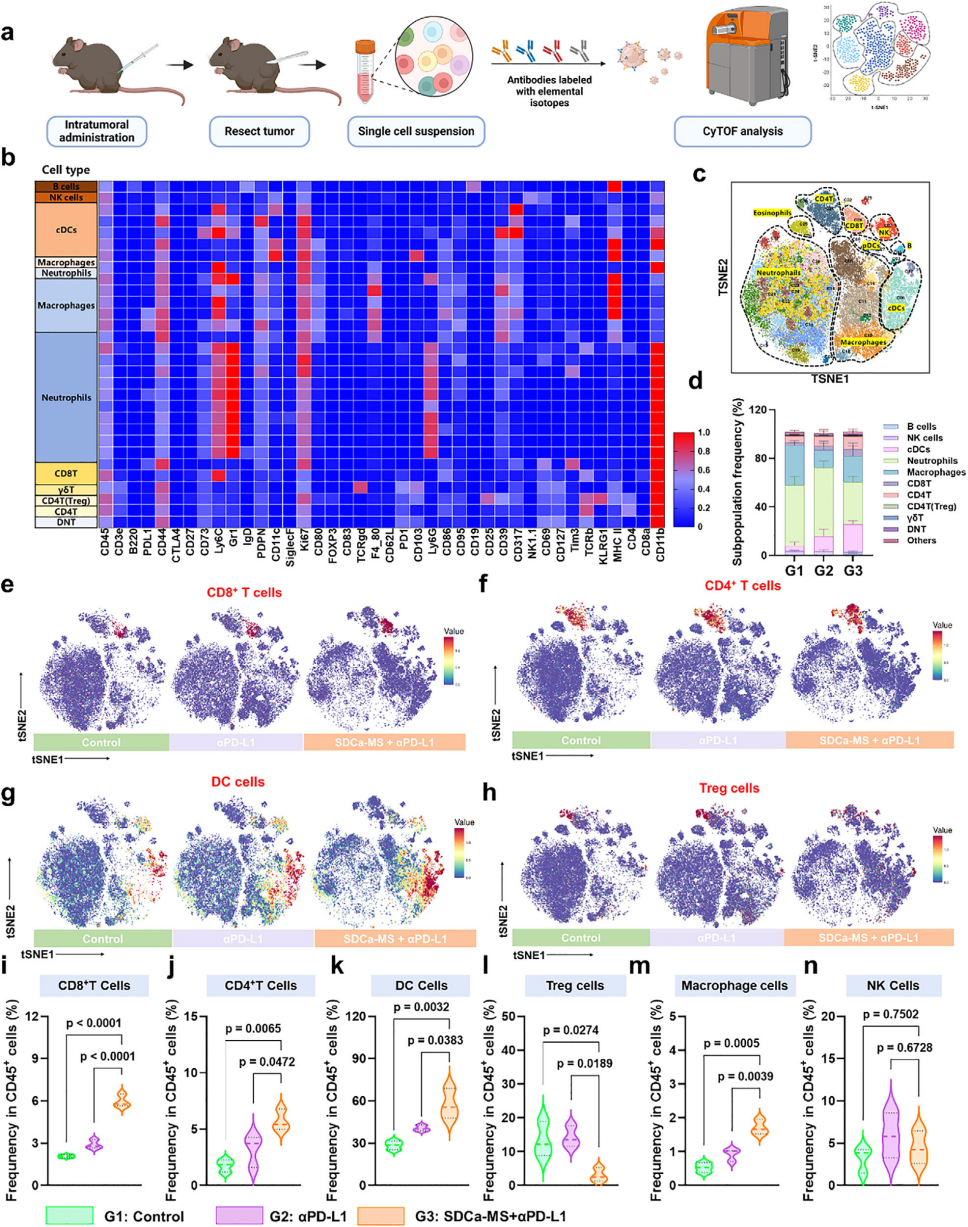
CyTOF Reveals Comprehensive Remodeling of the Tumor Immune Landscape. (a) Experimental design and analysis workflow for single‐cell CyTOF. (b) Heatmap showing the normalized expression of the 40 immune markers across the 27 identified immune cell clusters. (c) t‐SNE map of CD45^+^ tumor‐infiltrating immune cells, colored according to the 27 phenotypically distinct clusters identified across all samples. (d) Relative abundances of major tumor‐infiltrating immune cell populations identified by CyTOF analysis. (e–h) t‐SNE projections showing the distributions of CD8^+^ T cells (e), CD4^+^ T cells (f), DCs (g), and Tregs (h) across the treatment groups. (i–n) Quantification of CD8^+^ T cells (i), CD4^+^ T cells (j), DCs (k), Tregs (l), macrophages (m), and NK cells (n). Data are presented as mean ± SD (*n* = 3). Statistical analyses were performed as described in the Experimental Section. Exact p‐values are indicated in the corresponding panels.

### 2.8 Evaluation of Embolization Performance and Therapeutic Efficacy in an Orthotopic N1‐S1 HCC Model

To evaluate the embolization performance of SDCa‐MSs, a decellularized liver model was established to visualize microsphere distribution within the hepatic vascular network (Figure 6a). Fluorescence imaging showed that SDCa‐MSs efficiently penetrated distal hepatic vessels and were uniformly distributed throughout the vascular tree, with fluorescence signals confined to the vascular lumen, indicating effective intravascular embolization (Figure 6b). These results confirm the favorable embolization characteristics of SDCa‐MSs for transarterial delivery.

**FIGURE 6:**
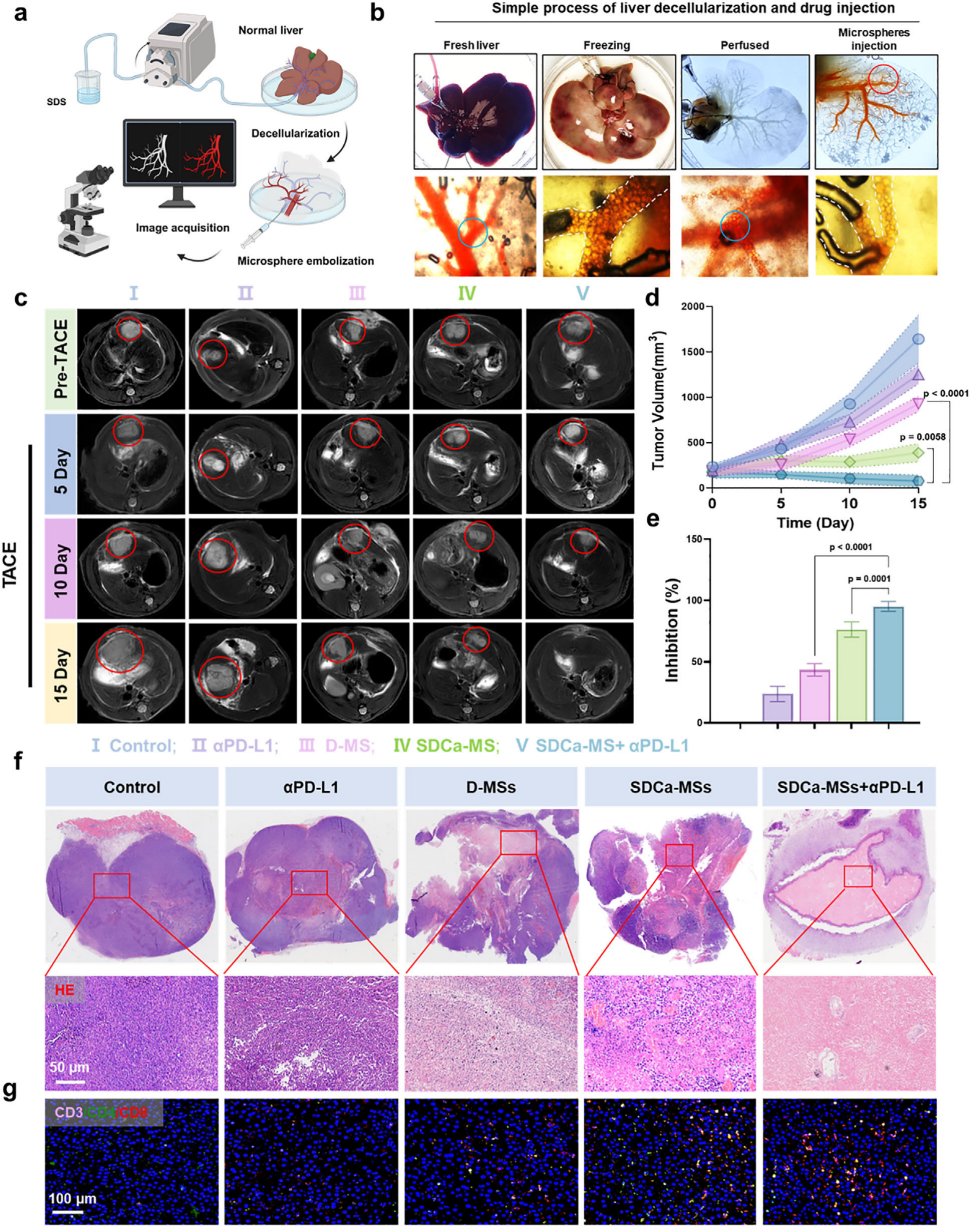
Evaluation of Embolization Performance and Therapeutic Efficacy in an Orthotopic N1‐S1 HCC Model. (a) Schematic illustration of the preparation of the decellularized liver model and evaluation of microsphere embolization. (b) Representative images showing liver decellularization and the intravascular distribution of microspheres within the hepatic vascular network. (c) Representative T2‐weighted MRI of orthotopic N1‐S1 tumor‐bearing rats following different treatments. (d) Tumor growth curves of orthotopic N1‐S1 tumor‐bearing rats after different treatments. (e) Tumor inhibition rates of orthotopic N1‐S1 liver tumors following different treatments. (f) Representative H&E‐stained tumor sections collected from orthotopic N1‐S1 tumor‐bearing rats after different treatments. (g) Representative immunofluorescence images showing tumor‐infiltrating CD3^+^, CD4^+^, and CD8^+^ T cells in tumor tissues from different treatment groups; nuclei were counterstained with DAPI. Data are presented as mean ± SD (*n* = 5). Statistical analyses were performed as described in the Experimental Section. Exact p‐values are indicated in the corresponding panels.

The therapeutic efficacy of SDCa‐MSs was subsequently evaluated in an orthotopic N1‐S1 HCC model using digital subtraction angiography (DSA)‐guided transarterial chemoembolization (Figures S39–S41). Magnetic resonance imaging (MRI) demonstrated progressive tumor regression following combination treatment with SDCa‐MSs and αPD‐L1, whereas tumors in the control groups continued to enlarge over time (Figure 6c). Quantitative analysis further confirmed that combination therapy achieved the greatest therapeutic efficacy, resulting in complete tumor eradication in two rats and an overall tumor inhibition rate of 93.8%, substantially exceeding those of D‐MSs (42.5%) and αPD‐L1 monotherapy (23.7%) (Figure 6d,e). Histopathological analyses confirmed extensive tumor necrosis in the combination therapy group (Figure 6f). Immunofluorescence staining showed reduced expression of the lactate transporters MCT1 and MCT4 following SDCa‐MSs treatment (Figure S42). As the principal effector cells mediating antitumor immunity and the primary targets of immune checkpoint blockade, T cells were selected for spatial validation by multiplex immunofluorescence in the orthotopic rat model. Multiplex immunofluorescence revealed markedly increased infiltration of CD3^+^, CD4^+^, and CD8^+^ T cells within tumor tissues (Figure 6g). Throughout the treatment period, body weight remained stable across all treatment groups (Figure S43). Histological examination of the heart, spleen, lung, and kidney revealed no obvious pathological abnormalities (Figure S44). Routine hematological parameters and liver function‐related biochemical indices likewise showed no significant abnormalities after SDCa‐MSs treatment (Figure S45). Together, these results demonstrate that SDCa‐MSs combined with αPD‐L1 achieve potent therapeutic efficacy with favorable biosafety in an orthotopic HCC model.

## 3 Conclusions

In summary, we developed a multifunctional embolic microsphere (SDCa‐MSs) that enables coordinated regulation of tumor lactate homeostasis through extracellular proton buffering and intracellular lactate retention. By simultaneously targeting both extracellular and intracellular metabolic abnormalities, SDCa‐MSs reverse lactate‐driven metabolic adaptation, restore chemosensitivity, and induce calcium dyshomeostasis‐associated mitochondrial dysfunction, ultimately triggering ICD and remodeling the tumor immune microenvironment. These coordinated effects promote both innate and adaptive antitumor immunity, markedly enhance the therapeutic efficacy of immune checkpoint blockade, and generate robust local and systemic antitumor responses. Taken together, this work establishes coordinated regulation of tumor acid–base homeostasis as an effective strategy for integrating metabolic intervention with TACE‐based immunotherapy and provides a promising biomaterial platform for improving the treatment of HCC.

## 4 Experimental Section

### 4.1 Materials

Ammonium carbonate [(NH_4_)_2_CO_3_], anhydrous calcium chloride (CaCl_2_), sodium octanoate, Syr, and DOX were obtained from Aladdin (Shanghai, China). Dichloromethane (DCM) and lactic acid were obtained from Sigma–Aldrich (St. Louis, MO, USA). PLGA (lactide/glycolide ratio = 50:50, MW = 33 000 Da) was obtained from Daigang Biomaterial Co., Ltd. (Shandong, China).

### 4.2 Cell Culture

The HCC cell lines Hepa1‐6 and N1‐S1 were obtained from the American Type Culture Collection (ATCC, Manassas, VA, USA). Hepa1‐6 cells were cultured in Dulbecco's Modified Eagle Medium (DMEM; Gibco, Carlsbad, CA, USA) supplemented with 10% fetal bovine serum (FBS; Gibco, Carlsbad, CA, USA) and 1% (v/v) penicillin–streptomycin (Gibco, Carlsbad, CA, USA). N1‐S1 cells were cultured in Iscove's Modified Dulbecco's Medium (IMDM; ATCC, Manassas, VA, USA) supplemented with 10% FBS and 1% (v/v) penicillin–streptomycin. Cells were maintained in a humidified incubator at 37°C with 5% CO_2_. To mimic the acidic tumor microenvironment, 10 mM lactic acid (LA; Sigma–Aldrich, St. Louis, MO, USA) was added to the culture medium where indicated.

### 4.3 Animals and Tumor Models

#### 4.3.1 Animals

Male C57BL/6 mice (4–6 weeks old, approximately 20 g) and Sprague‐Dawley rats (6–8 weeks old, approximately 220 g) were obtained from Hangzhou Qizhen Experimental Animal Technology Co., Ltd. (Hangzhou, China; SCXK(Zhe)2022‐0005). All animal experiments were performed in accordance with the Guidelines for the Care and Use of Laboratory Animals and were approved by the Animal Ethics Committee of Lishui University (Approval No. 2024YD0057).

#### 4.3.2 Subcutaneous Mouse Tumor Model

Hepa1‐6 cells (5 × 10^6^ cells in 100 µL of PBS) were subcutaneously injected into the right hindlimb of C57BL/6 mice to establish the subcutaneous tumor model.

#### 4.3.3 Bilateral Mouse Tumor Model

Hepa1‐6 cells (5 × 10^6^ cells in 100 µL of PBS) were subcutaneously injected into the right flank of C57BL/6 mice to establish the primary tumors (Day 0). Three days later, the same number of cells was injected into the left flank to establish the distant tumors.

#### 4.3.4 Orthotopic Rat Liver Tumor Models

Sprague‐Dawley rats were anesthetized, and N1‐S1 cells (1 × 10^7^ cells in 200 µL of PBS) were injected beneath the capsule of the left hepatic lobe to establish orthotopic liver tumors.

### 4.4 Human HCC Specimens and Immunofluorescence Analysis

Human HCC and paired adjacent non‐tumor liver tissue specimens were obtained from patients who underwent surgical resection at Lishui Central Hospital. The study was conducted in accordance with the ethical standards and approved by the Ethical Review Committee of Lishui Central Hospital (Approval No. 2025(1)‐140‐01). Immunofluorescence staining was performed for LDHA (Proteintech, Wuhan, China), MCT1 (Abcam, Shanghai, China), and MCT4 (Abcam, Shanghai, China) using the EnVision G|2 Doublestain System according to the manufacturer's instructions.

### 4.5 Bioinformatics Analysis

Clinical data and protein expression profiles were retrieved from The Cancer Genome Atlas (TCGA) database (www.tcga‐data.nci.nih.gov/tcga). Survival analysis was conducted using the Kaplan‐Meier method, and survival rates were compared utilizing the log‐rank test. To evaluate the association between lactate metabolism and therapeutic response, the gene expression dataset GSE104580 was downloaded from the Gene Expression Omnibus (GEO) database. Patients were classified as responders and non‐responders according to the original study design. DEGs between the two groups were identified using the limma package in R (version 4.3.1). Genes with an adjusted *p*‐value < 0.05 and |log_2_ fold change| > 1 were considered significantly differentially expressed. Functional enrichment analysis of DEGs was performed using the clusterProfiler package to identify significantly enriched KEGG pathways. Volcano plots and other data visualizations were generated using ggplot2.

### 4.6 Quantification of Intracellular and Extracellular Lactate Levels

Intracellular and extracellular lactate concentrations were quantified using a commercial Lactic Acid Assay Kit (Nanjing Jiancheng Bioengineering Institute, Nanjing, China). Hepa1‐6 cells were seeded in six‐well plates at a density of 2 × 10^5^ cells per well and cultured overnight. Cells were then treated with free CaCO_3_ (15 µg·mL^−1^), Syr (1 µg·mL^−1^), or their combination for 24 h. After treatment, the conditioned medium was collected and centrifuged to remove cellular debris for extracellular lactate measurement. The cells were subsequently harvested, washed twice with PBS, and lysed by sonication on ice. Cell lysates were centrifuged at 8000 rpm for 10 min at 4°C, and the supernatants were collected for intracellular lactate determination. Lactate concentrations in both the conditioned medium and cell lysates were measured according to the manufacturer's instructions. Absorbance was recorded at 530 nm using a microplate reader (InfiniteM200 PRO; Tecan, Männedorf, Switzerland), and lactate concentrations were calculated using a standard calibration curve.

### 4.7 Measurement of Extracellular and Intracellular pH

Intracellular pH was measured using the pH‐sensitive fluorescent probe BCECF‐AM (Beyotime Biotechnology, Shanghai, China). Hepa1‐6 cells were seeded in black‐walled, clear‐bottom 96‐well plates at a density of 8 × 10^3^ cells per well and cultured overnight. Cells were treated under the same conditions as described above. Subsequently, BCECF‐AM was added to each well at a final concentration of 1 µM and incubated at 37°C for 30 min in the dark. After washing with PBS, fluorescence images were acquired using a confocal laser scanning microscope (CLSM, STELLARIS 5, Leica, Wetzlar, Germany). Intracellular pH values were determined according to the manufacturer's instructions using the Intracellular pH Calibration Buffer Kit (Beyotime Biotechnology, Shanghai, China), and calculated from a calibration curve. For extracellular pH measurement, conditioned medium was collected after treatment, and the pH was measured immediately using a calibrated pH meter.

### 4.8 Cellular Uptake

Hepa1‐6 cells were seeded in six‐well plates and pretreated with 10 mM lactic acid overnight. Cells were then treated with CaCO_3_, Syr, or their combination for 24 h, followed by incubation with DOX (1 µg·mL^−1^) for an additional 12 h. After treatment, the cells were fixed with 4% paraformaldehyde for 20 min at 4°C and stained with DAPI (Solarbio, Beijing, China) for 15 min at 37°C to visualize the nuclei. Intracellular localization of DOX was observed using a CLSM (STELLARIS 5; Leica, Wetzlar, Germany). Cellular uptake of DOX was further quantified by flow cytometry using a FACSCanto II flow cytometer (BD Biosciences, San Diego, CA, USA).

### 4.9 Measurement of Intracellular Ca^2+^ Levels

Intracellular Ca^2+^ levels were measured using the calcium‐sensitive fluorescent probe Fluo‐4 AM (Beyotime Biotechnology, Shanghai, China). Hepa1‐6 cells were seeded in 12‐well plates at a density of 1 × 10^5^ cells per well and cultured overnight. Cells were treated under the same conditions as described above. After treatment, the cells were incubated with 5 µM Fluo‐4 AM at 37°C for 30 min in the dark according to the manufacturer's instructions. Following PBS washing to remove excess probe, intracellular Ca^2+^ fluorescence was observed using a CLSM. Quantitative analysis of intracellular Ca^2+^ levels was performed by flow cytometry using a FACSCanto II flow cytometer.

### 4.10 Measurement of Mitochondrial Membrane Potential

Mitochondrial membrane potential was evaluated using a JC‐1 assay kit (Beyotime Biotechnology, Shanghai, China). Cells were treated under the same conditions as described above. After treatment, the cells were washed with PBS and incubated with JC‐1 working solution at 37°C for 20 min according to the manufacturer's instructions. The cells were then washed with JC‐1 staining buffer (1×), and fluorescence images were acquired using a CLSM. JC‐1 aggregates and monomers were detected in the red and green fluorescence channels, respectively. Mitochondrial membrane potential was quantified as the ratio of red‐to‐green fluorescence intensity using ImageJ software (National Institutes of Health, Bethesda, MD, USA).

### 4.11 Western Blot Analysis

Hepa1‐6 cells were seeded in six‐well plates at a density of 2 × 10^5^ cells per well and cultured overnight. Total cellular proteins were extracted using RIPA lysis buffer, and protein concentrations were determined using a BCA Protein Assay Kit (Beyotime Biotechnology, Shanghai, China). Equal amounts of protein were mixed with 1× SDS‐PAGE loading buffer, denatured at 95°C for 5 min, separated by SDS‐PAGE, and transferred onto polyvinylidene fluoride (PVDF) membranes using a Trans‐Blot Turbo Transfer System (Bio‐Rad, Hercules, CA, USA). The membranes were blocked with 5% non‐fat milk in TBST for 2 h at room temperature and incubated overnight at 4°C with primary antibodies against ATP2B4 (1:2,000, Abcam, Shanghai, China), Calpain‐1 (1:1000, Proteintech, Wuhan, China), and β‐actin (1:10 000, ABclonal, Wuhan, China). After washing three times with TBST, the membranes were incubated with an HRP‐conjugated secondary antibody (1:10 000, Beyotime Biotechnology, Shanghai, China) for 1 h at room temperature. Protein bands were visualized using an enhanced chemiluminescence (ECL) detection system (Bio‐Rad, Hercules, CA, USA) and imaged with an iBright FL1000 imaging system (Thermo Fisher Scientific, Waltham, MA, USA).

### 4.12 TEM Observation of Mitochondrial Ultrastructure

Hepa1‐6 cells were seeded in six‐well plates and cultured overnight. Cells were treated under the same conditions as described above. After treatment, the cells were collected by centrifugation and fixed in 2.5% glutaraldehyde overnight at 4°C. The samples were then post‐fixed with 1% osmium tetroxide for 1 h at room temperature, dehydrated through a graded ethanol series, and embedded in epoxy resin. Ultrathin sections were prepared, stained with uranyl acetate and lead citrate, and examined using a transmission electron microscope (Hitachi, Tokyo, Japan) to evaluate mitochondrial ultrastructural alterations.

### 4.13 Intracellular ATP Measurement

Hepa1‐6 cells were seeded in six‐well plates and pretreated with 10 mM lactic acid overnight. After treatment, intracellular ATP levels were determined using an ATP Assay Kit (Beyotime Biotechnology, Shanghai, China) according to the manufacturer's instructions. Luminescence was measured using a luminometer, and ATP levels were quantified based on the recorded luminescence intensity.

### 4.14 Mitochondrial ROS Measurement

Mitochondrial ROS levels were measured using the MitoSOX Red Mitochondrial Superoxide Indicator (Thermo Fisher Scientific, Waltham, MA, USA). Cells were treated under the same conditions as described above. After treatment, the cells were incubated with 5 µM MitoSOX Red at 37°C for 20 min in the dark according to the manufacturer's instructions. The cells were then washed with PBS to remove excess probe, and mitochondrial ROS fluorescence was quantified using a FACSCanto II flow cytometer.

### 4.15 Preparation and Characterization of CaNPs

CaNPs with an average diameter of approximately 100 nm were synthesized using a gas diffusion method as previously reported [32]. Briefly, 150 mg of CaCl_2_ was dissolved in 50 mL of ethanol in a conical flask. The flask was then placed in a vacuum desiccator containing 5 g of (NH_4_)_2_CO_3_ and maintained at 37°C for 24 h to allow gas‐phase diffusion and CaCO_3_ precipitation. To obtain hydrophobic CaNPs, 1 mL of sodium n‐octanoate solution (3 mg·mL^−1^ in ethanol) was added to 5 mL of suspension (5 mg·mL^−1^ in ethanol), followed by sonication in a water bath for 20 min. The resulting nanoparticles were collected by centrifugation at 14 800 rpm, washed repeatedly with ethanol, and finally redispersed in dichloromethane (DCM) for subsequent experiments. The morphology of the CaNPs was characterized using a TEM (HT7800; Hitachi, Tokyo, Japan), and the particle size distribution was determined using a Zetasizer Nano ZS (ZSU3100, Malvern Panalytical, Malvern, UK).

### 4.16 Preparation of SDCa‐MSs

SDCa‐MSs were prepared from CaNPs and PLGA using a water‐in‐oil‐in‐water (W/O/W) double emulsion solvent evaporation method. Briefly, 1 mL of DOX solution (2 mg·mL^−1^) was added to 2 mL of dichloromethane (DCM) containing Syr (2 mg·mL^−1^), PLGA (80 mg·mL^−1^), and CaNPs (5 mg·mL^−1^). The mixture was emulsified using a probe sonicator (SCIENTZ VORTEX‐5, Scientz, Ningbo, China) at 40 kHz for 5 min in an ice‐water bath to obtain the primary (W/O) emulsion. The resulting emulsion was subsequently dispersed into 15 mL of 1 wt.% poly(vinyl alcohol) (PVA) aqueous solution under sonication to form the secondary (W/O/W) emulsion, followed by magnetic stirring at 37°C for 4 h to allow complete evaporation of DCM. The microspheres were collected by centrifugation at 5000 rpm, washed three times with deionized water to remove residual PVA, and stored at 4°C until further use. SCa‐MSs, DCa‐MSs, and Ca‐MSs were prepared using the same procedure by omitting Syr and/or DOX during the formulation process. Similarly, S‐MSs, D‐MSs, and SD‐MSs were prepared following the same protocol in the absence of CaNPs.

### 4.17 Characterization of SDCa‐MSs

The morphology and elemental distribution of the SDCa‐MSs were characterized using SEM equipped with energy‐dispersive X‐ray spectroscopy (EDS) (SU8600, Hitachi, Tokyo, Japan). The UV–vis absorption spectra of the SDCa‐MSs were recorded over a wavelength range of 250–650 nm using a UV–vis spectrophotometer (UV1900i, Shimadzu, Kyoto, Japan). The chemical structures of the microspheres were further analyzed by FTIR spectroscopy (IRSpirit‐T, Shimadzu, Kyoto, Japan).

### 4.18 Storage Stability

The prepared microspheres were stored at 4°C. At predetermined time points over a period of 7 days, the microsphere morphology was observed by optical microscopy, and the size distribution was determined by image analysis using ImageJ software (National Institutes of Health, Bethesda, MD, USA).

### 4.19 Measurement of the EE and DL Capacity

EE and DL of SD‐MSs and SDCa‐MSs were determined using UV–vis spectrophotometry. Briefly, 50 mg of freshly prepared SDCa‐MSs were collected by centrifugation, and the supernatant containing the unencapsulated drugs was retained for analysis. The concentrations of free DOX and Syr were determined using a UV–vis spectrophotometer at 488 and 257 nm, respectively. EE was calculated as the percentage of encapsulated drug relative to the initial amount of drug added during preparation, whereas DL was calculated as the percentage of encapsulated drug relative to the total mass of the drug‐loaded microspheres according to Equations (1) and (2).

(1) $$\mathrm{EE}\;\left(\%\right)=\frac{Mass\;of\;encapsulated\;drug}{Mass\;of\;initially\;added\;drug}*100\%\;$$

(2) $$\begin{array}{c} \mathrm{DL}\;\left(\%\right)=\frac{Mass\;of\;encapsulated\;drug}{Total\;mass\;of\;drug-loaded\;microspheres}*100\% \end{array}$$

### 4.20 In Vitro Drug Release

The in vitro release profiles of Syr and DOX from SD‐MSs and SDCa‐MSs were evaluated using the dialysis method. Briefly, equal amounts of SD‐MSs or SDCa‐MSs were suspended in 20 mL of PBS (pH 6.0 or 7.4) and sealed in dialysis bags (MWCO: 3.5 kDa). The dialysis bags were incubated at 37°C with gentle shaking at 100 rpm. At predetermined time points, 1 mL of the release medium was collected and replaced with an equal volume of fresh PBS pre‐equilibrated to the same pH. The released DOX and Syr were quantified by fluorescence spectroscopy and UV–vis spectrophotometry, respectively, using standard calibration curves. The cumulative release percentages were calculated after correction for the drug removed during each sampling and expressed relative to the total amount of encapsulated drug.

### 4.21 Evaluation of Acid‐Neutralizing Capacity

The acid‐neutralizing capacity of SD‐MSs and SDCa‐MSs was evaluated using a pH microelectrode (pH‐1^ST^; PreSens, Regensburg, Germany). Briefly, SD‐MSs or SDCa‐MSs were dispersed in PBS adjusted to pH 6.0 or 6.5. The pH was monitored over time using the pH microelectrode according to the manufacturer's instructions.

### 4.22 Cell Cytotoxicity Assay

The cytotoxicity of the various microsphere formulations was evaluated using the MTT assay (Beyotime Biotechnology, Shanghai, China). Hepa1‐6 cells were seeded in 96‐well plates at a density of 8 × 10^3^ cells per well and incubated overnight in the presence of lactate. The cells were subsequently treated with different concentrations of microspheres for 24 h at 37°C. Thereafter, 20 µL of MTT solution (5mg·mL^−1^) was added to each well, followed by incubation for an additional 4 h. The culture medium was then removed, and 150 µL of dimethyl sulfoxide was added to dissolve the formazan crystals. Absorbance was measured at 490 nm using a microplate reader. Cell viability was calculated according to Equation (3):

(3) $$\mathrm{Cell}\;\mathrm{viability}\;\left(\%\right)=\frac{A1}{A0}*100\%$$

where A_1_ and A_0_ represent the mean absorbance values of the experimental and control groups, respectively.

Cell viability was further assessed using a Live/Dead Cell Staining Kit (Beyotime Biotechnology, Shanghai, China). Following treatment with the indicated microsphere formulations, the cells were washed three times with PBS and stained with calcein‐AM and propidium iodide (PI) for 30 min in the dark according to the manufacturer's instructions. Fluorescence images were acquired using a CLSM.

### 4.23 Apoptosis Detection

Cell apoptosis was evaluated using an Annexin V‐FITC/PI Apoptosis Detection Kit (Beyotime Biotechnology, Shanghai, China). Hepa1‐6 cells were seeded in six‐well plates and incubated overnight in the presence of lactate. The cells were subsequently treated with the indicated microsphere formulations for 24 h, harvested, and resuspended in 100 µL of binding buffer. Apoptotic cells were stained with Annexin V‐FITC and PI according to the manufacturer's instructions and analyzed by flow cytometry. The data were analyzed using FlowJo software (version 10.8.1; FlowJo LLC, Ashland, OR, USA).

### 4.24 In Vitro Evaluation of ICD

ICD was assessed by measuring the exposure or release of its hallmark biomarkers, including CRT, HMGB1, and extracellular ATP. For CRT staining, Hepa1‐6 cells were treated with the indicated microsphere formulations for 12 h, fixed with 4% paraformaldehyde, and incubated with an anti‐CRT primary antibody (1:200, Proteintech, Wuhan, China) overnight at 4°C. After washing with PBS, the cells were incubated with the corresponding fluorescent secondary antibody (1:1000) and DAPI (1:800), followed by observation using a CLSM. HMGB1 release was quantified using an HMGB1 ELISA kit (IBL International, Hamburg, Germany). Culture supernatants were collected after treatment and centrifuged to remove cellular debris, and HMGB1 levels were determined according to the manufacturer's instructions. Extracellular ATP release was measured using an ATP Assay Kit (Beyotime Biotechnology, Shanghai, China). Culture supernatants were collected after treatment and centrifuged to remove cellular debris. ATP levels were determined according to the manufacturer's instructions using a luminometer.

### 4.25 Co‐Culture of Tumor Cells, BMDCs and Splenic Lymphocytes

Bone marrow‐derived dendritic cells (BMDCs) were generated from C57BL/6 mice. Briefly, bone marrow cells were harvested by flushing the femurs and tibias with sterile PBS and cultured in complete RPMI‐1640 medium supplemented with GM‐CSF (20 ng·mL^−1^, PeproTech, Suzhou, China). The culture medium was replaced every 2 days with fresh GM‐CSF‐containing medium to induce differentiation into immature BMDCs. Splenic lymphocytes were isolated from the spleens of C57BL/6 mice using Lympholyte‐M density‐gradient medium (Solarbio, Beijing, China) according to the manufacturer's instructions. Hepa1‐6 cells were treated with the indicated microsphere formulations for 24 h and subsequently co‐cultured with immature BMDCs and splenic lymphocytes. After 48 h of co‐culture, the cells were collected and stained with the indicated fluorochrome‐conjugated antibodies. Dendritic cells (DCs) were identified using FITC‐conjugated anti‐mouse CD11c, PE‐conjugated anti‐mouse CD80, and APC‐conjugated anti‐mouse CD86 antibodies. T‐cell subsets were analyzed using APC‐conjugated anti‐mouse CD3, PE‐conjugated anti‐mouse CD4, and FITC‐conjugated anti‐mouse CD8α antibodies. All antibodies were obtained from BioLegend (Beijing, China) and used at a dilution of 1:100. All stained cell populations were analyzed by flow cytometry. The concentrations of tumor necrosis factor‐α (TNF‐α) and interferon‐γ (IFN‐γ) in the culture supernatants were quantified using ELISA kits according to the manufacturer's instructions.

### 4.26 In Vivo Retention and Biodistribution

Indocyanine green (ICG)‐loaded microspheres (ICG‐MSs and ICG@Ca‐MSs) were prepared using the same procedure as that used for SDCa‐MSs. Tumor‐bearing mice received intratumoral injections of the ICG‐loaded microspheres and were anesthetized for fluorescence imaging at 6, 24, 48, and 72 h post‐injection using an IVIS Lumina III in vivo imaging system (PerkinElmer, Waltham, MA, USA). After the final imaging time point, the mice were euthanized, and the major organs (heart, liver, spleen, lungs, kidneys, and tumor) were harvested for ex vivo fluorescence imaging. Fluorescence intensity was quantified using the IVIS Lumina III imaging system.

### 4.27 In Vivo Antitumor Efficacy

To evaluate the antitumor efficacy in vivo, Hepa1‐6 tumor‐bearing mice with a tumor volume of approximately 100 mm^3^ were randomly assigned to six groups and intratumorally injected with saline, D‐MSs, SCa‐MSs, SD‐MSs, DCa‐MSs, or SDCa‐MSs. Body weight and tumor volume were recorded every 3 days. Tumor volume was calculated according to Equation (4):

(4) $$\mathrm{Tumor}\;\mathrm{volume}=\mathrm{length}\;\times \;\mathrm{widt}h^{2}\;/2$$

At the end of the treatment, tumors were harvested, fixed, and processed for H&E, TUNEL, and Ki‐67 immunohistochemical staining to evaluate histopathological changes, apoptosis, and tumor cell proliferation, respectively.

### 4.28 In Vivo Evaluation of Tumor Acidity Neutralization

To evaluate the in vivo tumor acidity‐neutralizing capacity of SDCa‐MSs, Hepa1‐6 tumor‐bearing mice were randomly assigned to four groups receiving saline, Ca‐MSs, SD‐MSs, or SDCa‐MSs. Following treatment, the mice were intravenously injected with the pH‐sensitive fluorescent probe 5‐(and‐6)‐carboxy SNARF‐4F (1 nmol per mouse). After 30 min, the mice were euthanized, and the tumors were immediately excised and bisected for fluorescence imaging using an IVIS Lumina III imaging system. Fluorescence images were acquired at emission wavelengths of 580 and 640 nm with an excitation wavelength of 500 nm. A calibration curve was established based on the fluorescence intensity ratio at 580 and 640 nm (I_580_/I_640_) across different pH values, and the intratumoral pH was calculated accordingly.

### 4.29 In Vivo ROS Detection

Fresh tumor tissues were embedded in optimal cutting temperature (OCT) compound and cryosectioned into 8 µm‐thick sections. The sections were incubated with 5 µM dihydroethidium (DHE, Beyotime Biotechnology, Shanghai, China) at 37°C for 30 min in the dark, followed by washing with PBS three times. The nuclei were counterstained with DAPI, and fluorescence images were acquired using a CLSM.

### 4.30 Intratumoral Ca^2+^ Detection

Frozen tumor sections were incubated with Fluo‐3 AM (Beyotime Biotechnology, Shanghai, China) according to the manufacturer's instructions to detect intracellular Ca^2+^ levels. After washing with PBS, the sections were incubated in the dark at 37°C for 30 min, followed by counterstaining with DAPI to visualize nuclei. Fluorescence images were acquired using a CLSM.

### 4.31 In Vivo Evaluation of ICD

Tumor tissues were fixed in 4% paraformaldehyde, embedded in paraffin, and sectioned into 4 µm‐thick sections. Following deparaffinization, rehydration, and heat‐induced antigen retrieval, the sections were blocked with 5% BSA for 1 h at room temperature and incubated overnight at 4°C with primary antibodies against CRT (1:200, Beyotime Biotechnology, Shanghai, China) and HMGB1 (1:200, Beyotime Biotechnology, Shanghai, China). After washing with PBS, the sections were incubated with secondary antibody (1:500, Beyotime Biotechnology, Shanghai, China) for 1 h. The nuclei were counterstained with DAPI, and fluorescence images were captured using a CLSM.

### 4.32 Evaluation of Immune Microenvironment Modulation in Vivo

The murine tumor models and treatment protocols were established as described above. At the designated time point, tumors and spleens were harvested, mechanically dissociated, and enzymatically digested to obtain single‐cell suspensions. The cells were stained with the indicated fluorochrome‐conjugated antibodies according to the manufacturers' instructions and analyzed by flow cytometry. Splenic mature DCs (CD11c^+^CD80^+^CD86^+^) were analyzed to evaluate systemic immune activation. Tumor‐infiltrating immune cell populations, including total DCs (CD11c^+^MHC‐II^+^), mature DCs (CD11c^+^MHC‐II^+^CD86^+^), CD4^+^ T cells (CD3^+^CD4^+^), CD8^+^ T cells (CD3^+^CD8^+^), Tregs (CD4^+^Foxp3^+^CD25^+^), M1 macrophages (CD11b^+^F4/80^+^CD86^+^), and M2 macrophages (CD11b^+^F4/80^+^CD206^+^) were quantified. Intratumoral levels of TNF‐α and IFN‐γ were quantified using commercial ELISA kits according to the manufacturers' instructions. Data acquisition was performed using a BD FACSCanto II flow cytometer, and data were analyzed with FlowJo software version 10.8.1.

### 4.33 In Vivo Antitumor Efficacy of SDCa‐MSs Combined With αPD‐L1 Therapy

To further evaluate the therapeutic efficacy of SDCa‐MSs in combination with αPD‐L1, antitumor studies were performed using a bilateral Hepa1‐6 tumor model. Mice were randomly assigned to four groups receiving PBS, αPD‐L1, SDCa‐MSs, or SDCa‐MSs + αPD‐L1. αPD‐L1 was administered intravenously at a dose of 1 mg·kg^−1^ (Bio X Cell, Lebanon, NH, USA). Body weight and tumor volume were recorded throughout the treatment period, and the mice were euthanized on day 15. Intratumoral levels of TNF‐α and IFN‐γ were quantified using commercial ELISA kits according to the manufacturers' instructions. Tumor immune cell infiltration was analyzed following the procedures described above.

### 4.34 Mass Cytometry (CyTOF) Analysis

Single‐cell suspensions were prepared from tumor tissues as described above. Immune cells were stained with a 40‐metal‐conjugated antibody panel (Table S2) according to the manufacturer's instructions. Data were acquired using a Helios mass cytometer (Fluidigm, South San Francisco, CA, USA) by detecting metal isotope signals corresponding to the conjugated antibodies. Dimensionality reduction was performed using t‐distributed stochastic neighbor embedding (t‐SNE), followed by PhenoGraph‐based clustering to identify distinct immune cell populations. Data were further analyzed using FlowJo software (version 10.8.1; FlowJo LLC, Ashland, OR, USA).

### 4.35 Preparation of Decellularized Liver and Evaluation of the Embolization Performance of SDCa‐MSs

Decellularized livers were prepared from livers harvested from male Sprague‐Dawley rats. Briefly, the rats were euthanized, and the livers were excised, placed in sealed containers, and stored at −80°C overnight. The livers were subsequently decellularized according to a previously reported protocol [33]. After thawing at room temperature, the livers were perfused through the portal vein with distilled water for 30 min, followed by 4% Triton X‐100 (Beyotime Biotechnology, Shanghai, China) for 3 h and 0.5% sodium dodecyl sulfate (SDS) solution for 8 h at a constant flow rate of 7 mL·min^−1^ until the tissues became translucent. The resulting decellularized livers were used to evaluate the embolization performance of SDCa‐MSs during TACE.

### 4.36 Evaluation of TACE Treatment

The establishment of the orthotopic rat HCC model for evaluating the TACE treatment efficacy of SDCa‐MSs is described in Section 4.3. After the tumor size reached approximately 100 mm^3^, the N1‐S1 tumor–bearing rats were randomly assigned to five treatment groups (*n* = 5/group): G1, Control; G2, αPD‐L1 (1 mg·kg^−1^); G3, TACE with D‐MSs; G4, TACE with SDCa‐MSs; G5, TACE with SDCa‐MSs + αPD‐L1 (80 mg of microspheres in 0.2 mL of lipiodol per rat). For the TACE procedure, the rats were anesthetized, and a midline laparotomy was performed to expose the hepatic artery and gastroduodenal artery. The gastroduodenal artery was isolated and ligated distally, and a catheter was introduced into the hepatic artery through the gastroduodenal artery using a custom‐made puncture needle. Hepatic angiography was performed under digital subtraction angiography (DSA) guidance to identify the tumor‐feeding arteries. A suspension containing 80 mg of D‐MSs or SDCa‐MSs in 0.2 mL of lipiodol was subsequently administered under DSA guidance until marked reduction or disappearance of tumor vascular staining was observed. Post‐embolization angiography was performed to confirm successful occlusion of the tumor‐feeding vessels. The catheter was then withdrawn, the gastroduodenal artery was ligated, and the abdominal incision was closed. Tumor progression was monitored using a 3.0 T Ingenia MRI system (Philips, Amsterdam, The Netherlands).

### 4.37 Histological, Immunohistochemical, and Immunofluorescence Analyses

On day 15 after treatment, the rats were euthanized, and tumor tissues were harvested and fixed in 4% paraformaldehyde, embedded in paraffin, and sectioned. Histopathological evaluation was performed by H&E staining. Immunohistochemical staining was performed to evaluate the expression of MCT1 and MCT4. To evaluate tumor‐infiltrating T cells, immunofluorescence staining was performed using antibodies against CD3, CD4, and CD8, followed by imaging with a CLSM.

### 4.38 In Vivo Safety Analysis

Following treatment, the rats were euthanized, and the major organs were harvested, fixed in 4% paraformaldehyde, embedded in paraffin, and sectioned for histological evaluation. H&E staining was performed to assess potential treatment‐related histopathological changes. Whole blood was collected for routine hematological analysis using a fully automated hematology analyzer (TEK8500 VET; TECOM, China) and serum biochemical analysis using a blood biochemistry analyzer (MNCHIP, China).

### 4.39 Statistical Analysis

All quantitative data are presented as the mean ± standard deviation (SD) unless otherwise indicated. Raw data were checked for data integrity before analysis, and no data points were excluded as outliers. Statistical analyses were performed using GraphPad Prism 10.0 (GraphPad Software, San Diego, CA, USA) and R version 4.3.1, as appropriate. ImageJ software (National Institutes of Health, Bethesda, MD, USA) was used for image‐based quantitative analysis. Comparisons between two groups were performed using an unpaired two‐tailed Student's t‐test, whereas multiple‐group comparisons were analyzed by one‐way or two‐way ANOVA, as appropriate, followed by Tukey's multiple‐comparisons test. For IC_50_ determination, drug concentrations were log‐transformed, and dose‐response curves were fitted by nonlinear regression. Differences in gene expression between tumor and normal tissues were assessed using the unpaired Wilcoxon rank‐sum test, and Kaplan‐Meier survival curves were compared using the log‐rank test. All statistical tests were two‐sided, and *p* < 0.05 was considered statistically significant. The sample size (n) for each experiment is indicated in the corresponding figure legends, with exact p‐values provided in the corresponding figures where applicable.

## Author Contributions

M.C.: conceptualization, data curation, formal analysis, writing – original draft, and funding acquisition. X.C.: methodology, data curation, formal analysis, writing – original draft, and funding acquisition. B.Y.: data curation and investigation. H.X.: methodology, validation, and data curation. X.S.: methodology and data curation. H.Q.: data curation and investigation. H.H.: methodology and investigation. R.W.: software and formal analysis. F.Q.: methodology and formal analysis. B.W.: methodology and investigation. Y.L.: validation, supervision, writing – review and editing, resources, and software. G.S.: project administration, writing – review and editing, funding acquisition, resources, and software. J.J.: supervision, writing – review and editing, visualization, project administration, and conceptualization.

## Funding

This work was supported by the National Natural Science Foundation of China (82272090, 82472088); the “Pioneer” and “Leading Goose” R&D Program of Zhejiang (2023C03062); the Natural Science Foundation of Zhejiang Province (LLSSZ24H180001); the Zhejiang Medicine and Health Science and Technology Project (2024KY561, 2025KY494).

## AI Statement

Generative AI tools were used solely for language polishing and grammar correction. All AI‐assisted edits were reviewed and verified by the authors, who take full responsibility for the manuscript.

## Conflicts of Interest

The authors declare no conflicts of interest.

## Supporting information




**Supporting File**: advs78484‐sup‐0001‐SuppMat.docx.

## Data Availability

The data that support the findings of this study are available from the corresponding author upon reasonable request.
