# Supplementary material for: Lactate Homeostasis Disruption Potentiates TACE‐Immunotherapy in Hepatocellular Carcinoma
Source: Adv Sci (Weinh). 2026 Oct 28:e78484. Online ahead of print. doi: 10.1002/advs.78484 (PMC13715671; doi:10.1002/advs.78484)
Supplement: Supplementary file 1 [file ADVS-9999-e78484-s001.docx]

**Supplemental Information**

**Lactate Homeostasis Disruption Potentiates TACE-Immunotherapy in Hepatocellular Carcinoma**

*Minjiang Chen^#^, Xiaoxiao Chen^#^, Baozhu Yang, Hui Xu, Xueting Sun, Hongkai Qi, Huilin Hu, Ronghua Wu, Fengkai Qiu, Bing Wu, Yu Luo*, Gaofeng Shu*, and Jiansong Ji**

Table S1. List of abbreviations

| **Abbreviation** | **Full Name** |
| --- | --- |
| αPD-L1 | Anti-programmed death-ligand 1 antibody |
| ANOVA | Analysis of variance |
| APC | Antigen-presenting cell |
| ATP | Adenosine triphosphate |
| ATP2B4 | ATPase plasma membrane Ca^2+^ transporting 4 |
| BMDCs | Bone marrow-derived dendritic cells |
| Ca-MSs | CaNPs-loaded microspheres |
| CaNPs | Calcium carbonate nanoparticles |
| CLSM | Confocal laser scanning microscopy |
| CRT | Calreticulin |
| CyTOF | Cytometry by time-of-flight |
| DAMPs | Damage-associated molecular patterns |
| DC | Dendritic cell |
| DCa-MSs | Doxorubicin/CaNPs-loaded microspheres |
| DEGs | Differentially expressed genes |
| DHE | Dihydroethidium |
| DL | Drug loading |
| DMEM | Dulbecco's modified Eagle medium |
| D-MSs | Doxorubicin-loaded microspheres |
| DOX | Doxorubicin |
| DSA | Digital subtraction angiography |
| EE | Encapsulation efficiency |
| ELISA | Enzyme-linked immunosorbent assay |
| FBS | Fetal bovine serum |
| FTIR | Fourier transform infrared spectroscopy |
| GEO | Gene Expression Omnibus |
| GM-CSF | Granulocyte-macrophage colony-stimulating factor |
| HCC | Hepatocellular carcinoma |
| H&E | Hematoxylin and eosin |
| HMGB1 | High mobility group box 1 |
| ICD | Immunogenic cell death |
| ICG | Indocyanine green |
| IF | Immunofluorescence |
| IFN-γ | Interferon gamma |
| IHC | Immunohistochemistry |
| KEGG | Kyoto Encyclopedia of Genes and Genomes |
| LDHA | Lactate dehydrogenase A |
| MCT | Monocarboxylate transporter |
| MCT1 | Monocarboxylate transporter 1 (SLC16A1) |
| MCT4 | Monocarboxylate transporter 4 (SLC16A3) |
| MRI | Magnetic resonance imaging |
| MSs | Microspheres |
| MTT | 3-(4,5-Dimethylthiazol-2-yl)-2,5-diphenyltetrazolium bromide |
| NK | Natural killer cell |
| OCT | Optimal cutting temperature |
| PBS | Phosphate-buffered saline |
| PLGA | Poly(lactic-co-glycolic acid) |
| PVA | Poly(vinyl alcohol) |
| ROS | Reactive oxygen species |
| SCa-MSs | Syrosingopine/CaNPs-loaded microspheres |
| SD | Standard deviation |
| SD-MSs | Syrosingopine/Doxorubicin-loaded microspheres |
| SDCa-MSs | Syrosingopine/Doxorubicin/CaNPs-loaded microspheres |
| SDS-PAGE | Sodium dodecyl sulfate-polyacrylamide gel electrophoresis |
| SEM | Scanning electron microscopy |
| Syr | Syrosingopine |
| TACE | Transarterial chemoembolization |
| TBST | Tris-buffered saline with Tween 20 |
| TCGA | The Cancer Genome Atlas |
| TEM | Transmission electron microscopy |
| TME | Tumor microenvironment |
| TNF-α | Tumor necrosis factor alpha |
| Treg | Regulatory T cell |
| t-SNE | t-distributed stochastic neighbor embedding |
| TUNEL | Terminal deoxynucleotidyl transferase dUTP nick-end labeling |

Table S2. Heavy metal-labeled antibody panel for CyTOF analysis.

Figure S1. Intracellular pH levels following different treatments. Data are presented as mean ± SD (n = 3). Statistical significance was assessed using one-way ANOVA followed by Tukey's multiple-comparisons test. Exact p values are indicated in the corresponding panel.

Figure S2. Representative confocal laser scanning microscopy images of Fluo-4-stained Hepa1-6 cells after different treatments.

Figure S3. Mean fluorescence intensity ratio of JC‐1 aggregates and JC‐1 monomers. Data are presented as mean ± SD (n = 3). Statistical significance was assessed using one-way ANOVA followed by Tukey's multiple-comparisons test. Exact p values are indicated in the corresponding panel.

Figure S4. Relative ATP levels of Hepa1-6 cells after different treatments. Data are presented as mean ± SD (n = 3). Statistical significance was assessed using one-way ANOVA followed by Tukey's multiple-comparisons test. Exact p values are indicated in the corresponding panel.

Figure S5. Detection of ROS levels by flow cytometry.

Figure S6. (A) SEM image and (B) particle size distribution of SD-MSs.

Figure S7. Particle size stability of SD-MSs and SDCa-MSs during the 7-day storage period. Data are presented as mean ± SD (n = 3). Statistical significance was assessed using one-way ANOVA followed by Tukey's multiple-comparisons test.

Figure S8. Energy-Dispersive X-ray Spectroscopy (EDS) of SDCa-MSs.

Figure S9. DL% of Syr and DOX in SD-MSs and SDCa-MSs. Data are presented as mean ± SD (n = 3). Statistical significance was assessed using one-way ANOVA followed by Tukey's multiple-comparisons test.

Figure S10. *In vitro* release profiles of Syr and DOX from SD-MSs under different pH conditions (pH 6.0 and 7.4). Data are presented as mean ± SD (n = 3). Statistical significance was assessed using one-way ANOVA followed by Tukey's multiple-comparisons test.

Figure S11. Time-dependent acid-neutralizing capacities of SDCa-MSs and SD-MSs at an initial pH of 6.0. Data are presented as mean ± SD (n = 3). Statistical significance was assessed using one-way ANOVA followed by Tukey's multiple-comparisons test.

Figure S12. Cell viability of Hepa1-6 cells in different groups. Data are presented as mean ± SD (n = 6). Statistical significance was assessed using one-way ANOVA followed by Tukey's multiple-comparisons test. Exact p values are indicated in the corresponding panel.

Figure S13. (A) Apoptosis assessed by flow cytometry in Hepa1-6 cells following various treatments. (B) Quantitative measurement of apoptosis rates in Hepa1-6 cells following various treatments. The total apoptosis rate was calculated as the sum of the early and late apoptosis rates. Data are presented as mean ± SD (n = 3). Statistical significance was assessed using one-way ANOVA followed by Tukey's multiple-comparisons test. Exact p values are indicated in the corresponding panel.

Figure S14. (A) Fluorescence microscopy images of live/dead staining, where green indicates live cells and red indicates dead cells. (B) Quantitative analysis of live and dead cells based on live/dead staining. Data are presented as mean ± SD (n = 3). Statistical significance was assessed using one-way ANOVA followed by Tukey's multiple-comparisons test.

Figure S15. (A) Representative CLSM images of cell-surface CRT exposure after different treatments. (B) Quantification of CRT exposure. (C) ELISA-based analysis of HMGB1 release in the culture supernatant. (D) Measurement of extracellular ATP release in the culture supernatant following different treatments. Data are presented as mean ± SD (n = 3). Statistical significance was assessed using one-way ANOVA followed by Tukey's multiple-comparisons test. Exact p values are indicated in the corresponding panel.

Figure S16. Schematic diagram of the ternary co-culture process.

Figure S17. Flow cytometry plots and quantitative analysis of mature BMDCs (CD11c^+^CD80^+^CD86^+^) in the ternary co-culture system. Data are presented as mean ± SD (n = 3). Statistical significance was assessed using one-way ANOVA followed by Tukey's multiple-comparisons test. Exact p values are indicated in the corresponding panel.

Figure S18. Flow cytometry analysis of CD3^+^CD4^+^ helper T cells and CD3^+^CD8^+^ cytotoxic T cells in the ternary co-culture system. Data are presented as mean ± SD (n = 3). Statistical significance was assessed using one-way ANOVA followed by Tukey's multiple-comparisons test. Exact p values are indicated in the corresponding panel.

Figure S19. Quantification of pro-inflammatory cytokines TNF-α (A) and IFN-γ (B) in the supernatants, measured by ELISA. Data are presented as mean ± SD (n = 3). Statistical significance was assessed using one-way ANOVA followed by Tukey's multiple-comparisons test. Exact p values are indicated in the corresponding panel.

Figure S20. *In vivo* distribution of microspheres in mice. (A) *In vivo* imaging illustrating the distribution of different formulations at various time points. (B) *Ex vivo* imaging of isolated organs from different groups after 72 h. (C) Semi-quantitative analysis of fluorescence intensity in tumors *in vivo*. (D) Semi-quantitative analysis of fluorescence intensity in isolated organs *ex vivo*. Data are presented as mean ± SD (n = 5). Statistical significance was assessed using one-way ANOVA followed by Tukey's multiple-comparisons test. Exact p values are indicated in the corresponding panel.

Figure S21. Tumor lactate content following different treatments. Data are presented as mean ± SD (n = 5). Statistical significance was assessed using one-way ANOVA followed by Tukey's multiple-comparisons test. Exact p values are indicated in the corresponding panel.

Figure S22. Representative fluorescence images and quantitative analysis of intratumoral Ca^2+^ detected by Fluo-3 AM staining (green). Nuclei were counterstained with DAPI (blue). Scale bar: 100 μm. Data are presented as mean ± SD (n = 5). Statistical significance was assessed using one-way ANOVA followed by Tukey's multiple-comparisons test. Exact p values are indicated in the corresponding panel.

Figure S23. Representative fluorescence images and quantitative analysis of DHE-stained tumor sections following different treatments. ROS-associated fluorescence is shown in red, and nuclei were counterstained with DAPI (blue). Scale bar: 100 μm. Data are presented as mean ± SD (n = 5). Statistical significance was assessed using one-way ANOVA followed by Tukey's multiple-comparisons test. Exact p values are indicated in the corresponding panel.

Figure S24. Representative fluorescence images and quantitative analysis of nuclear HMGB1 (green) and CRT (red) in tumor tissues collected from mice following different treatments. Nuclei were counterstained with DAPI (blue). Scale bar: 100 μm. Data are presented as mean ± SD (n = 5). Statistical significance was assessed using one-way ANOVA followed by Tukey's multiple-comparisons test. Exact p values are indicated in the corresponding panel.

Figure S25. Histological analysis of tumor sections: Hematoxylin and eosin (H&E) staining, TUNEL staining, and Ki-67 staining of tumor sections from the different treatment groups.

Figure S26. (A-B) Representative flow cytometry plots (A) and quantitative analysis (B) of tumor-infiltrating CD11c^+^MHC-II^+^ DCs. (C) Quantitative analysis of tumor-infiltrating CD11c^+^MHC-II^+^CD86^+^ mature DCs. Data are presented as mean ± SD (n = 3). Statistical significance was assessed using one-way ANOVA followed by Tukey's multiple-comparisons test. Exact p values are indicated in the corresponding panel.

Figure S27. Quantitative analysis of splenic mature DCs (CD11c^+^CD80^+^CD86^+^) following different treatments. Data are presented as mean ± SD (n = 3). Statistical significance was assessed using one-way ANOVA followed by Tukey's multiple-comparisons test. Exact p values are indicated in the corresponding panel.

Figure S28. Quantitative analysis of CD3^+^CD4^+^ (A) and CD3^+^CD8^+^ (B) T cells in different treatment groups. Data are presented as mean ± SD (n = 3). Statistical significance was assessed using one-way ANOVA followed by Tukey's multiple-comparisons test. Exact p values are indicated in the corresponding panel.

Figure S29. Flow cytometric (A) and quantitative (B) analysis of Tregs in tumors from different treatment groups. Data are presented as mean ± SD (n = 3). Statistical significance was assessed using one-way ANOVA followed by Tukey's multiple-comparisons test. Exact p values are indicated in the corresponding panel.

Figure S30. Quantitative analysis of tumor-infiltrating M1 macrophages in different treatment groups. Data are presented as mean ± SD (n = 3). Statistical significance was assessed using one-way ANOVA followed by Tukey's multiple-comparisons test. Exact p values are indicated in the corresponding panel.

Figure S31. Flow cytometric (A) and quantitative (B) analysis of M2 macrophages in tumors from different treatment groups. Data are presented as mean ± SD (n = 3). Statistical significance was assessed using one-way ANOVA followed by Tukey's multiple-comparisons test. Exact p values are indicated in the corresponding panel.

Figure S32. (A-B) ELISA analysis of tumor samples to determine IFN-γ (A) and TNF-α (B) levels. Data are presented as mean ± SD (n = 3). Statistical significance was assessed using one-way ANOVA followed by Tukey's multiple-comparisons test. Exact p values are indicated in the corresponding panel.

Figure S33. (A) Primary tumor growth curve, tumor weights and images during the treatment period. (B) Distant tumor growth curve, tumor weights and images during the treatment period. Data are presented as mean ± SD (n = 5). Statistical significance was assessed using one-way ANOVA followed by Tukey's multiple-comparisons test. Exact p values are indicated in the corresponding panel.

Figure S34. Flow cytometric analysis of M2 macrophages in distant tumors after different treatments. Data are presented as mean ± SD (n = 5). Statistical significance was assessed using one-way ANOVA followed by Tukey's multiple-comparisons test. Exact p values are indicated in the corresponding panel.

Figure S35. Single-cell CyTOF analysis. t-SNE plots showing the clustering and distribution of major immune cell subsets across different treatment groups.

Figure S36. (A) t-SNE visualization of B cells across different treatment groups. (B) Quantitative analysis of the percentage of B cells in each treatment group. Data are presented as mean ± SD (n = 3). Statistical significance was assessed using one-way ANOVA followed by Tukey's multiple-comparisons test. Exact p values are indicated in the corresponding panel.

Figure S37. t-SNE visualization of M1 macrophages across different treatment groups.

Figure S38. t-SNE visualization of NK cells across different treatment groups.

Figure S39. Schematic illustration of the TACE treatment protocol in N1-S1 tumor-bearing rats.

Figure S40. Surgical procedure for TACE in rats: (a) Tumor formation; (b) Identification and isolation of the artery; (c) Arterial ligation; (d) Arterial cannulation.

Figure S41. DSA-guided embolization of SDCa-MSs. Representative DSA images acquired before (pre-TACE) and after (post-TACE) embolization. Pre-TACE imaging demonstrates successful perfusion of the contrast agent into the tumor-feeding arteries, whereas post-TACE imaging shows marked contrast agent reflux and disappearance of tumor vascular opacification, indicating successful embolization.

Figure S42. Representative immunofluorescence images of MCT1 and MCT4 expression in tumor tissues from different treatment groups. Nuclei were counterstained with DAPI.

Figure S43. Body weight changes in N1-S1 tumor-bearing rats throughout the experimental period. Data are presented as mean ± SD (n = 5). Statistical significance was assessed using one-way ANOVA followed by Tukey's multiple-comparisons test.

Figure S44. Representative H&E staining images of the heart, spleen, lung, and kidney.

Figure S45. Hematological and liver function analyses following treatment with SDCa-MSs. Data are presented as mean ± SD (n = 5). Statistical significance was assessed using one-way ANOVA followed by Tukey's multiple-comparisons test.

Figure S46. Flow cytometry gating strategies for immune cell subsets. (A) Representative gating strategy for tumor-infiltrating DCs, identified as CD11c^+^MHC-II^+^ cells and further characterized by CD86 expression. (B) Representative gating strategy for tumor-infiltrating T-cell subsets, including CD3^+^CD8^+^ cytotoxic T cells and CD3^+^CD4^+^ helper T cells. (C) Representative gating strategy for Tregs, identified as CD4^+^Foxp3^+^CD25^+^ cells. (D) Representative gating strategy for tumor-associated macrophage (TAM) subsets, including M1-like TAMs (CD11b^+^F4/80^+^CD86^+^) and M2-like TAMs (CD11b^+^F4/80^+^CD206^+^).
